# Environmentally sensitive photosensitizers enable targeted photodynamic ablation of Gram-positive antibiotic resistant bacteria

**DOI:** 10.7150/thno.84187

**Published:** 2023-06-26

**Authors:** Sam Benson, Alex Kiang, Charles Lochenie, Navita Lal, Syam Mohan P. C. Mohanan, Gareth O. S. Williams, Kevin Dhaliwal, Bethany Mills, Marc Vendrell

**Affiliations:** 1 Centre for Inflammation Research, The University of Edinburgh, Edinburgh EH16 4TJ, UK.; 2 IRR Chemistry Hub, Institute for Regeneration and Repair, The University of Edinburgh, Edinburgh EH16 4UU, UK.

**Keywords:** photodynamic therapy, antimicrobial resistance, probes, biofilms, theranostic agents

## Abstract

Bacterial infections remain among the biggest challenges to human health, leading to high antibiotic usage, morbidity, hospitalizations, and accounting for approximately 8 million deaths worldwide every year. The overuse of antibiotics and paucity of antimicrobial innovation has led to antimicrobial resistant pathogens that threaten to reverse key advances of modern medicine. Photodynamic therapeutics can kill bacteria but there are few agents that can ablate pathogens with minimal off-target effects.

**Methods:** We describe nitrobenzoselenadiazoles as some of the first environmentally sensitive organic photosensitizers, and their adaptation to produce theranostics with optical detection and light-controlled antimicrobial activity. We combined nitrobenzoselenadiazoles with bacteria-targeting moieties (i.e., glucose-6-phosphate, amoxicillin, vancomycin) producing environmentally sensitive photodynamic agents.

**Results:** The labelled vancomycin conjugate was able to both visualize and eradicate multidrug resistant Gram-positive ESKAPE pathogens at nanomolar concentrations, including clinical isolates and those that form biofilms.

**Conclusion:** Nitrobenzoselenadiazole conjugates are easily synthesized and display strong environment dependent ROS production. Due to their small size and non-invasive character, they unobtrusively label antimicrobial targeting moieties. We envisage that the simplicity and modularity of this chemical strategy will accelerate the rational design of new antimicrobial therapies for refractory bacterial infections.

## Introduction

Bacterial infections are projected to cause over 10 million annual deaths globally by 2050 due to the rapid increase of antimicrobial resistance (AMR) 1. This rapid increase has been largely exacerbated by inappropriate use of antibiotics, leading to an urgent need for antimicrobial strategies for ESKAPE pathogens (*Enterococcus species, Staphylococcus aureus, Klebsiella pneumoniae, Acinetobacter baumannii, Pseudomonas aeruginosa and Enterobacter species*) that pose serious threats to human health 2. Only 12 new antibiotics have reached the market since 2017, with a small fraction representing major clinical benefits to existing treatments 3. Even for antibiotics against resistant infections (e.g., vancomycin), mechanisms disabling the action of the drugs are becoming more frequent 4. For example, *Enterococcus faecalis* shows vancomycin resistance via alterations of the D-ala-D-ala peptidoglycan region, which can be transferred to other *Enterococci* strains, such as *E. faecium* 5. As a result, vancomycin resistance is now found in >50% of *E. faecium* clinical isolates 6, leading to over 2-fold increased mortality rates when compared to non-resistant infections 7. Furthermore, Gram-positive bacteria that are exposed to vancomycin can lead to enhanced resistance (e.g., vancomycin-intermediate *S. aureus*) 8, which require the use of second-line antibiotics (e.g., tigecycline and daptomycin) with increased risk of toxic side effects 9.

Unlike conventional antibiotics, antimicrobial photodynamic therapy (aPDT) provides a means to kill bacterial pathogens without inducing further resistance 10. PDT combines non-toxic visible light, oxygen, and photosensitizers (PS) to ablate targeted cells (e.g., bacteria) without causing a major impact in neighboring cells. aPDT agents for Gram-positive bacteria are urgently needed, particularly for methicillin-resistant *S. aureus* (MRSA) and vancomycin-resistant *Enterococci* (VRE) as two of the most common drug-resistant pathogens in hospital-acquired infections. The groups of Ferreira-Strixino and Ip reported photodithazine and hypericin respectively as PS to clear MRSA infections 11,12, whilst Liu *et al.* showed that 5-amino levulinic acid could cause 5 log_10_ reduction in CFU counts of vancomycin-resistant *E. faecalis* after irradiation with 630 nm light 13. Other organic PS (e.g., curcumin, Rose Bengal) have been reported for the photodynamic ablation of pathogens 14,15; however, their inherent non-specificity is a major limitation for translational applications. One of the most conventional approaches to improve bacterial targeting is to embed PS within molecular structures that can bind to pathogens and increase their local concentration at infection sites. For instance, Ucuncu *et al*. described conjugates of polymyxin and methylene blue with selective uptake and phototoxicity in Gram-negative bacteria 16. More recently, Bourré *et al*. reported cell-penetrating peptides coupled to porphyrin for ablation of both Gram-negative and Gram-positive bacteria 17. Whereas these approaches can improve the target-to-background ratios of non-specific PS, they are still limited in that 1) large PS (e.g., porphyrins, phthalocyanines) can impair the molecular recognition properties of targeting ligands, and 2) most PS conjugates display off-target photosensitive behavior, and therefore washing steps or long incubation times are needed to generate singlet oxygen in infection microenvironments.

Our group and others have reported conjugatable PS for the preparation of targeted PDT agents 2,18,19. In this work, we focused on nitrobenzoselenadiazoles because of 1) their small size and neutral character, which make them suitable for the derivatization of targeting ligands (e.g., peptides) without altering the native recognition properties 20,21, and 2) their environmental sensitivity for increased production of singlet oxygen around bacterial membranes, maximizing the efficacy of aPDT under wash-free conditions. Using this strategy, we optimized the preparation of probe **6** as a benzoselenadiazole theranostic agent for fluorescence-based imaging of Gram-positive bacteria (to rapidly confirm presence of infection) and subsequent ablation using PDT. Finally, we demonstrated that this approach is effective for killing a wide range of drug-resistant Gram-positive pathogens and has potential application for the elimination of biofilms. The versatility of this chemical strategy and potential compatibility with multiple targeting ligands will speed up the design of phototheranostics for aPDT.

## Materials and Methods

### Chemical synthesis

Flash column chromatography was carried out using Silica 60A (particle size 35-70 μm) as the stationary phase. Thin layer chromatography (TLC) was performed using pre-coated silica gel plates (0.25 mm thick, 60 F254) and observed under UV light. All reagents were used without further purification unless otherwise stated. Spectroscopic experiments were carried out on a Synergy HT spectrophotometer, and data analysis was performed using GraphPad Prism 9.0. Reactions were monitored by HPLC-MS using a HPLC Agilent 1200 with a G1315B diode array detector and a 6110 quadrupole MS spectrometer with an electrospray ionization source. Data acquisition was performed with Agilent OpenLab. HRMS (ESI positive) were obtained in an LTQ-FT Ultra mass spectrometer. NMR spectra were recorded on a Bruker AVA 500 at 308 K. Chemical shifts (δ) are reported in ppm and coupling constants (J) are reported in Hertz (Hz). Multiplicities: s = singlet, d = doublet, t = triplet, dd = doublet doublets, ddd = double double doublet, dt = double triplet, q= quartet and m = multiplet. Full details of the procedures used for chemical synthesis and the characterization data are provided in the Supporting Information.

### Photophysical characterization and PDT equipment set-up

Singlet oxygen quantum yields (φ_Δ_) were calculated using previously described methods 18.

The aPDT setup utilized a light emitting diode (LED) with center wavelength of 470 nm and full width half maximum of 25 nm in a downward configuration. An aspheric condenser lens with a diameter of 50.8 mm and focal length of 32 mm was used to collimate the beam to ensure homogenous illumination over the sample. The output power of the LED was adjusted by a current controller connected to the LED using an M8 four pin connector. The LED was attached to one side of the translating lens mount using an adaptor, the lens tube and lens were connected on the other side of the translation mount. The LED was positioned so that a standard tissue culture plate (such as a 96-well Corning Costar™ 3596, used for all bacterial aPDT) could be housed 2 cm below the illumination set-up. An optical power meter and power head were used to measure the optical power to each well in the well-plate. The aperture of the power head was fixed at 0.6 cm diameter, which was equivalent to the diameter of a well in the well plate. The power mapping was performed by moving the sensor head on each well to measure the optical power. The irradiance was calculated as power per area of exposure in mW cm^-2^. Wells within the tissue culture plates with measured power 12±2 mW and corresponding irradiance 44±6 mW cm^-2^ were selected for experimentation, with samples assigned randomly to each well.

### Haemolysis assays

Blood was collected from healthy human volunteers following informed consent and ethical approval at the University of Edinburgh (21-EMREC-041). Erythrocytes isolated from peripheral blood were diluted to 1.4% (v/v) in PBS and incubated with compounds for 15 min prior to incubation with or without illumination (470 nm, 44 mW cm^-2^, 20 min). Following illumination, samples were centrifuged, and the absorbance values of the supernatants were measured at 415 nm (Synergy™ HTX Multi-Mode Microplate Reader) to determine the extent of haemolysis. Full haemolysis (positive control) was achieved by sonicating erythrocyte samples for 20 s and no haemolysis (negative control) was achieved with erythrocytes in PBS.

### Mammalian cell toxicity assays

The human keratinocyte cell line HaCaT (Cell Line Service; article 300493) was grown in Dulbecco's Modified Eagle Medium containing 10% fetal bovine serum, L-glutamine and Pen-Strep at 37 °C and 5% CO_2_. When reaching 80% confluency, cells (2×10^5^ cells mL^-1^) were seeded into 96-well plates and incubated at 37 °C and 5% CO_2_ for 24 h. Compounds were added to the cells followed by incubation in the dark at the indicated concentrations. Next, cells underwent illumination or were kept in the dark. All cells were then incubated in the dark at 37 °C for 24 h. MTT reagent was added and incubated at 37 °C and 5% CO_2_ for 4 h. Absorbance values were measured at 570/640 nm.

### Bacterial cell preparation

All bacterial strains were sourced from in-house strain collections, ATCC and the IHMA bacterial repository. The strains used in this study are *S. aureus* ATCC25923 (used in all *S. aureus* experiments unless otherwise stated); USA300, IHMA2190153; IHMA2043373. *S. epidermidis* IHMA1960576; *S. haemolyticus* IHMA2147409; *E. faecalis* ATCC51299; *E. faecium* IHMA 2024474; and *E. coli* ATCC25922. Single bacterial colonies were picked from Luria-Bertani (LB) agar plates and inoculated into LB broth, grown overnight at 37 °C under constant motion.

*Planktonic bacteria*. Overnight cultures were adjusted to OD_595_ 0.1 and incubated until mid-log phase (OD_595_ 0.4-0.8) under the same conditions. Bacteria concentrations were readjusted to the final concentration of OD_595_ 0.1 in sterile saline (0.9% NaCl). Bacteria were washed 3 times with sterile saline, followed by 1 min centrifugation at 10,600*g* and re-suspended in sterile saline for PDT and imaging experiments.

*Biofilms*. Overnight cultures of *S. aureus* were diluted to an OD_595_ 0.001 in Muller Hinton (MH) broth in 96-well plates. The plates were incubated at 37 °C without shaking for 24 h. Following which, the MH media was carefully removed, and biofilms were gently washed (by pipetting) in 0.9% NaCl, ready for further experimentation.

### Antimicrobial assays

*Planktonic bacteria*. Bacteria were prepared to an OD_595_ 0.1 in saline as outlined above. Bacteria were diluted 1:10 and compounds were added as appropriate in a total volume of 300 µL 0.9% NaCl for 10 min in the dark at r.t. Subsequently, bacteria requiring illumination were transferred into appropriate wells of a 96-well plate and illuminated within the LED set-up described above for 20 min. Control treatments were kept in the dark. Experiments were repeated a minimum of 3 times.

*Biofilms*. Compound **6** was added to biofilms at 250 µM and illuminated with the LED device at the indicated times and power. Where required, oxygen was bubbled into the biofilm media throughout the illumination (and equivalent duration in the dark) at a flow rate of 1 L min^-1^. Following treatment, biofilms were transferred to Precellys tubes and homogenized to disperse biofilms for CFU plating. For the quantification of PDT bacterial killing, serial dilutions in sterile saline were prepared. Each dilution was plated onto LB agar in triplicate and incubated overnight in a static incubator at 37 °C. CFU were counted the following day and presented as average CFU mL^-1^.

*Dark toxicity assays*. Bacterial overnight cultures were diluted to OD_595_ 0.001 in MH broth in 96-well plates. Compound **6** or unlabeled vancomycin were added as appropriate and bacterial growth was measured at 37 °C with shaking for 18 h using the microplate reader by absorbance at 600 nm. Conditions were plated in duplicate, and experiments were repeated 3 times.

*Vancomycin and teicoplanin MIC assay*s. LB agar plates were prepared with increasing concentrations of vancomycin or teicoplanin (0-100 µg mL^-1^). 20 µL of overnight bacterial cultures were pipetted onto the plates and allowed to dry. The plates were incubated at 37 ºC for 16 h and growth rates were recorded.

### Fluorescence confocal microscopy

Planktonic bacteria were grown and prepared in saline as outlined. Confocal 18-well imaging chamber slides (Ibidi 80826) were pre-coated with poly-D-lysine (0.1 mg mL^-1^). Each well was filled with 190 µL sterile saline and 10 µL of the bacterial suspension. Compounds were added at the indicated concentrations and confocal microscopy was performed on a Leica SP8 (HC PL APO CS2 63x 1.40 oil, HyD detectors) using 488 nm excitation. A minimum of 3 fields of view were captured per condition and repeated independently 3 times. Images were processed using the Leica Application Suite X (LAS X) software.

### Statistical analysis

Statistical analysis was carried out using Graphpad Prism. Statistical differences were determined via two-tailed unpaired t-tests or one-way ANOVA with comparisons based on a control column. CFU statistics were performed on log-transformed data. Independent experiments were performed at least 3 times, unless otherwise stated.

## Results and Discussion

### Chemical synthesis of nitrobenzoselenadiazole aPDT agents

**Figure 1:**
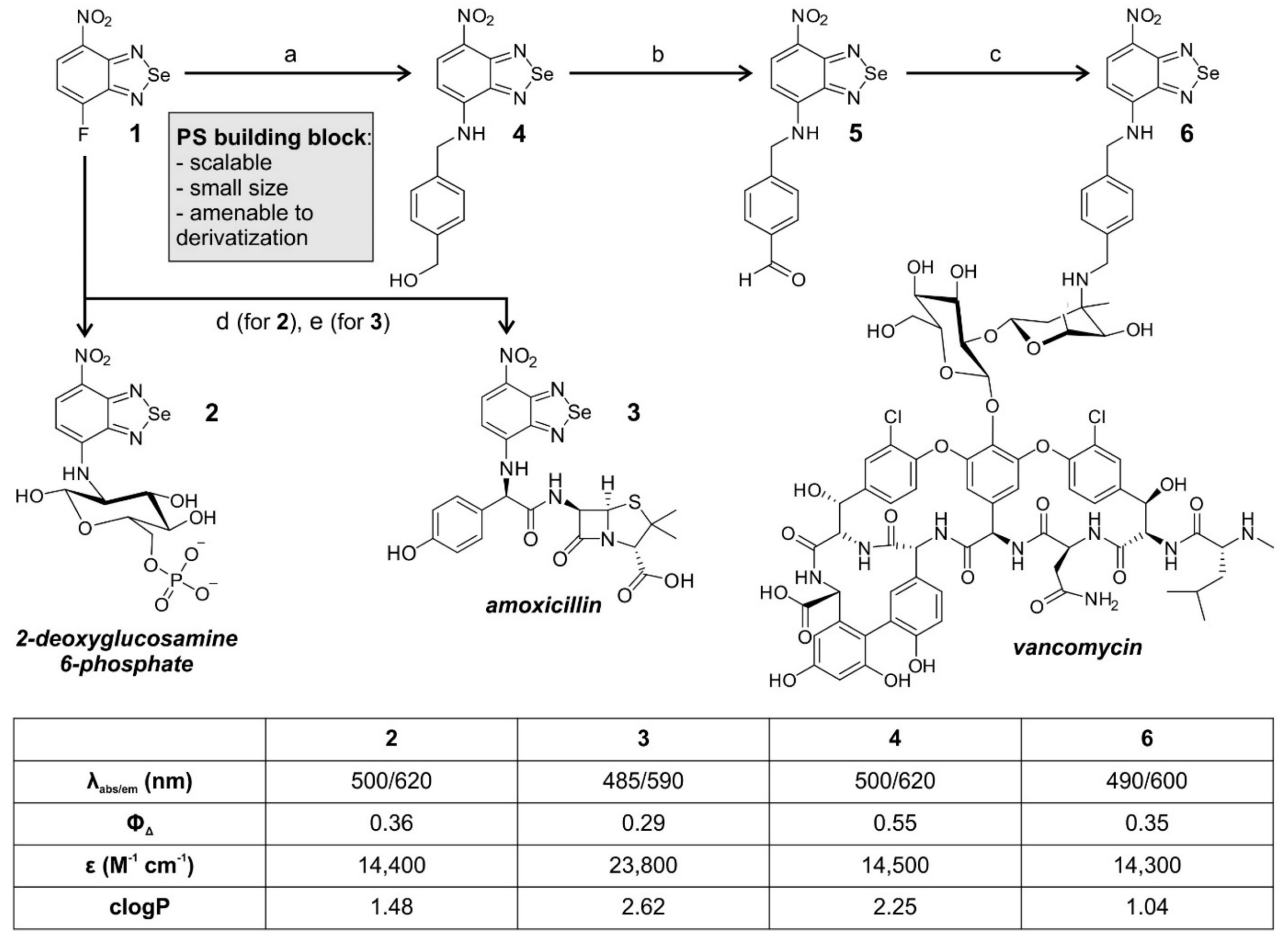
** Chemical synthesis of nitrobenzoselenadiazole-based antimicrobial theranostics.** Reaction conditions: a) 4-aminomethylbenzyl alcohol, DCM: EtOH (1:1), 2 h, r.t.; b) Dess-Martin periodinane, DCM, 4 h, r.t.; c) i. vancomycin HCl, DIPEA, DMF, 4 h, 55 °C; ii. NaBH_3_CN, TFA, MeOH, 2 h, r.t.; d) 4-deoxy-glucosamine-6-phosphate, NaHCO_3_, ACN:H_2_O, 24 h, r.t.; e) amoxicillin, DMSO, 16 h, r.t. Photophysical values measured in EtOH. Singlet oxygen generation quantum yields were determined using Rose Bengal as a reference 29.

We started the synthesis of nitrobenzoselenadiazole aPDT agents from the fluorinated precursor **1** (Figure 1). Compound **1** is a suitable building block for the preparation of targeted PS because it is amenable to nucleophilic substitution with biomolecules that contain reactive amines. Therefore, we decided to synthesize a small collection of probes by employing different amine-containing bacterial-targeting units, namely 2-deoxyglucosamine 6-phosphate, amoxicillin, and vancomycin. 2-deoxyglucosamine-6-phosphate is transported by universal hexose phosphate transporters (UHPT) into the cytoplasm of bacterial cells 22, whereas amoxicillin and vancomycin are pan-microbial and Gram-positive antibiotics, respectively. Amoxicillin is a β-lactam antibiotic that prevents peptidoglycan cross linking via interference with the enzymatic machinery involved, and vancomycin binds to the peptidoglycan chains in the cell envelope to block their synthesis 23.

The nitrobenzoselenadiazole conjugates with 2-deoxyglucosamine-6-phosphate (compound **2**, Figure 1) and amoxicillin (compound **3**, Figure 1) were prepared in a straightforward manner, using a single-step reaction between compound **1** and the corresponding amines. Both conjugates **2** and **3** were isolated in purities >95% (see Supporting Information for synthetic and characterization details). The preparation of the vancomycin derivative (compound **6**, Figure 1) required additional steps because vancomycin contains multiple reactive sites for derivatization. We selected the aminoglycan chain of vancomycin as the preferred conjugation site given that this position is not essential for binding to the bacterial cell wall 24, and that it has previously been modified with fluorescent dyes without negatively affecting the recognition of Gram-positive bacteria 25-28. The synthesis of compound **6** was designed in three steps. The incorporation of a 4-aminomethylbenzyl alcohol linker within the nitrobenzoselenadiazole scaffold for 1) selective conjugation through reductive amination, and 2) provision of an electron-donating amine group in the nitrobenzoselenadiazole, which is essential for the construction of a push-pull dipole and effective singlet oxygen production 18. After we reacted compound **1** with 4-aminomethylbenzyl alcohol to obtain compound **4**, we oxidized the benzylic alcohol using Dess-Martin periodinane to isolate the corresponding benzaldehyde **5** (Figure 1). Finally, the reaction between compound **5** and vancomycin via reductive amination yielded compound **6**, which was purified by semi-preparative HPLC and isolated with over 95% purity (see Supporting Information for synthetic and characterization details).

**Figure 2:**
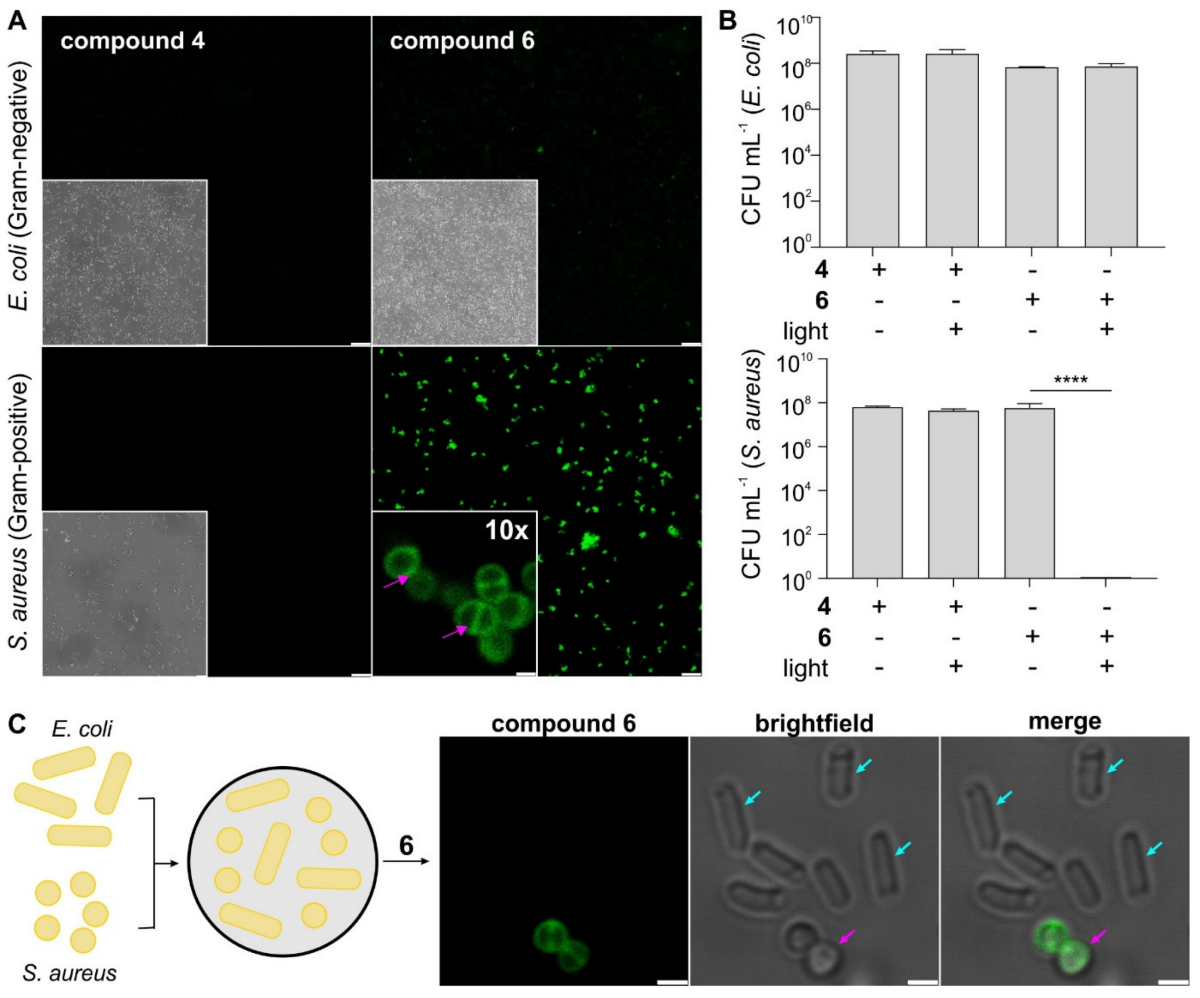
** Compound 6 shows selective labeling and phototoxicity in Gram-positive bacteria.** A) Representative brightfield and fluorescence confocal microscopy images of *S. aureus* and *E. coli* cells after incubation with non-targeted PS (compound **4**, 100 µM, green) and targeted PS (compound **6**, 100 µM, green). Excitation wavelength: 488 nm. Emission: 510-650 nm. Scale bar: 10 μm. High-magnification images of *S. aureus* incubated with compound **6**. Pink arrows indicate localization in the cell envelope and septum. Scale bar: 1 μm. B) Phototoxicity assays of compounds **4** and** 6** (5 μM) in *S. aureus* and *E. coli* with and without light irradiation (470 nm, 44 mW cm^-2^, 20 min). Data presented as means±SD (n=3). P values obtained from one-way ANOVA (**** for p<0.0001). C) Representative brightfield and fluorescence confocal microscopy images of *S. aureus* (pink arrows) and *E. coli* (blue arrows) cells after incubation with compound **6** (100 µM, green). Excitation wavelength: 488 nm. Emission: 510-650 nm. Scale bar: 1 μm.

We examined the photophysical properties of all bacterial-targeting conjugates (**2**, **3** and **6**) as well as the non-targeted PS **4** as a control. All benzoselenadiazole compounds showed similar excitation and emission wavelengths (~500 nm and 600 nm, respectively, Figure S1) and remarkable singlet oxygen generation under illumination with visible light (520 nm) with singlet oxygen quantum yields around 30% for all conjugates, confirming their potential application as theranostic probes for fluorescence imaging and PDT studies (Figure 1). Next, we evaluated the photosensitive potential of the three targeted conjugates (**2**, **3** and **6**) to kill bacterial cells. All three compounds were incubated with *S. aureus* and illuminated under the same experimental conditions. Compound **6** was highly phototoxic in *S. aureus*, whereas compounds **2** and **3** exhibited no phototoxicity (Figure 2 and Figure S2), potentially due to their limited accumulation in target cells. In view of the potential of compound **6** for aPDT, we decided to characterize its mode of action and further optimize its application as a theranostic agent for drug-resistant bacteria.

### Compound 6 localizes on the cell envelope and photoablates Gram-positive bacteria but not Gram-negative bacteria

One of the main advantages of nitrobenzoselenadiazoles over other PS is their small size and neutral character, which can facilitate the retention of activity after biomolecule derivatization. The bacterial-targeting moiety of compound **6** (i.e., vancomycin) is an antibiotic against Gram-positive bacteria with little effect on Gram-negative bacteria; therefore, we analyzed the selectivity of compound **6** against *S. aureus* and *E. coli* -as Gram-positive and Gram-negative bacterial strains, respectively, to evaluate the molecular impact of the nitrobenzoselenadiazole core.

First, we analyzed the cellular localization of compound **6** by fluorescence confocal microscopy. Compound **6** brightly labeled Gram-positive *S. aureus* but showed minimal fluorescence in Gram-negative *E. coli* under the same experimental conditions (Figure 2A and Figure S3), which was further exemplified in co-cultures of Gram-positive and Gram-negative bacteria (Figure 2C). Higher magnification microscopy images also confirmed the preferential accumulation of compound **6** in the cell envelope of *S. aureus* (Figure 2A and Figure S3), suggesting that the nitrobenzoselenadiazole did not impact the binding of vancomycin to the peptidoglycan moieties in bacterial cell walls. Similarly, the functionality of the vancomycin domain to act as an antibiotic over time was not impaired following conjugation of the PS (Figure S4). We also confirmed that the vancomycin moiety in compound **6** is essential for binding to bacterial cells, as the non-targeted compound **4** was unable to label Gram-positive or Gram-negative bacteria (Figure 2A).

Next, we determined the phototoxicity of compounds **4** and **6** against the same two bacterial strains by counting bacterial colonies grown following PDT treatment. We confirmed that the light illumination regime needed for photoactivation of compound **6** did not cause toxicity in *S. aureus* and *E. coli* (Figure S5) and observed complete eradication of *S. aureus* only when cells had been treated with compound **6** followed by illumination (Figure 2B and Figure S6). On the contrary, we did not detect any reduction in cell viability when Gram-positive bacteria had been treated with the compounds in the dark. Furthermore, there was no toxicity observed for an equimolar concentration of vancomycin under these conditions (Figure S7), further highlighting the efficacy and speed (i.e., under 30 min) of the aPDT treatment with compound **6**. In line with the selectivity profile observed in the fluorescence microscopy experiments, compound **6** showed no phototoxicity against the Gram-negative bacteria *E. coli* (Figure 2B). Together, this demonstrates the potential of compound **6** as a theranostic agent for selective imaging and photoablation of Gram-positive bacteria.

Finally, we measured the PDT minimum inhibitory concentration (PDT-MIC) of compound **6** as a Gram-positive PS, which was found to be around 300 nM for *S. aureus*. On the contrary, the non-targeted compound **4** showed no significant phototoxicity in bacterial cells, even at concentrations in the high micromolar range and much longer incubation times (Figure S8). Altogether, these results demonstrate that 1) some bacterial-targeting units (e.g., vancomycin) enable effective accumulation of nitrobenzodiazole PS in bacterial cells, 2) the coupling of nitrobenzoselenadiazoles to biomolecules can retain their cell selectivity profiles, and 3) constructs combining targeting moieties and nitrobenzoselenadiazoles (e.g., compound **6**) show remarkably lower working concentrations and incubation times than their individual counterparts (e.g., compound **4** and vancomycin).

### Compound 6 shows enhanced photosensitivity in hydrophobic environments

The fluorescence microscopy analysis of compound **6** in *S. aureus* showed the preferential accumulation of the nitrobenzoselenadiazole core in the cell envelopes of Gram-positive bacteria. In fact, subcellular localization plays a critical role in the capacity of PS to generate singlet oxygen concentrations that lead to effective cell ablation 30. For instance, the group of Beharry recently reported DNA-targeting PS based on the nuclear stain DAPI to produce ROS in close proximity to nucleic acids, which resulted in low MIC values against* E. coli* 31. Another recent example was reported by Calvaresi and co-workers, who described a concanavalin A-Rose Bengal conjugate to selectively ablate Gram-negative bacteria due to the selective binding of concanavalin to the lipopolysaccharide surface 32. Several groups have reported nitrobenzoxadiazoles as environmentally sensitive fluorophores 33-35. This feature has been exploited to design activatable probes that turn on in hydrophobic environments and display enhanced signal-to-noise ratios over 'always-on' fluorophores 36,37; however, the potential of nitrobenzoselenadiazoles as environmentally sensitive PS has not been studied to date. To address this point, we investigated the environmental sensitivity of the nitrobenzoselenadiazole unit in compound **6** and analyzed its capacity to generate singlet oxygen in different microenvironments.

**Figure 3:**
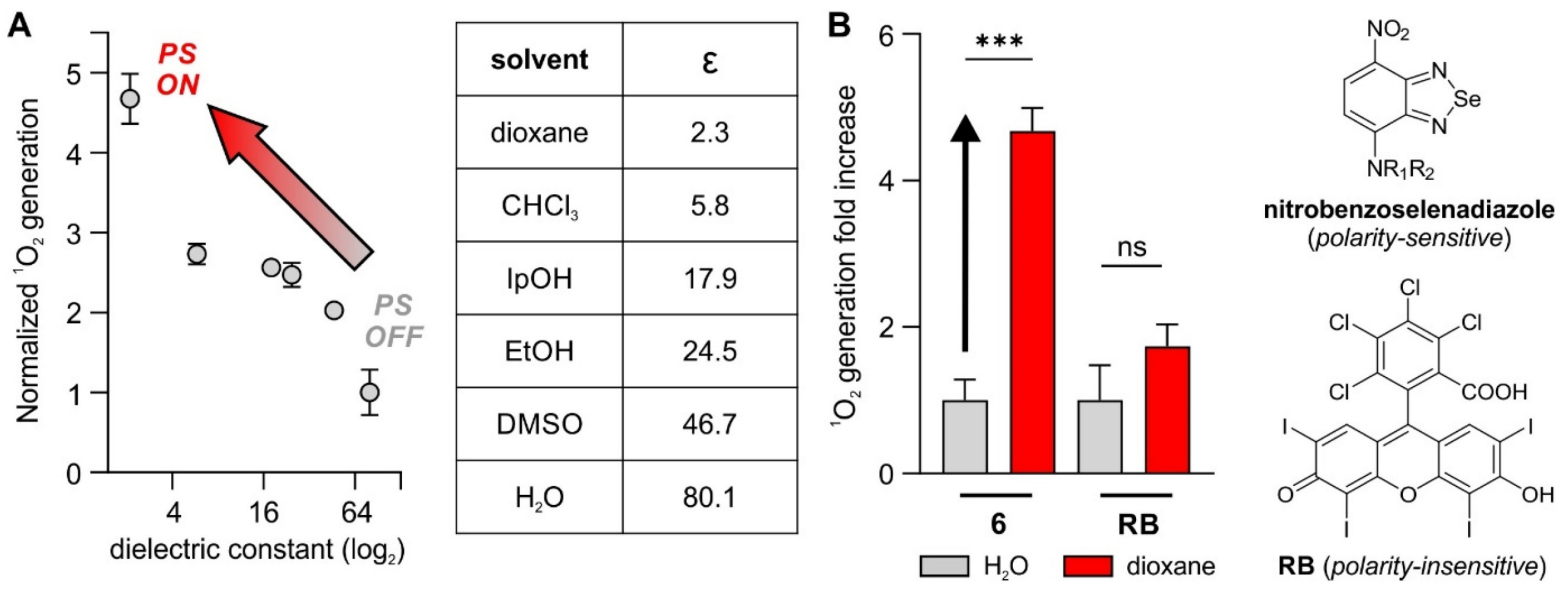
** Nitrobenzoselenadiazole PS show environment-dependent production of singlet oxygen.** A) Singlet oxygen generation, as measured by changes in absorbance of 1,3-diphenylisobenzofuran, from compound **6** in organic solvents with varying dielectric constants after light irradiation (520 nm, 0.5 mW, 60 s). Values presented as means±SD (n=3). B) Comparative analysis of singlet oxygen generation (measured by changes in the absorbance of 1,3-diphenylisobenzofuran at 410 nm) of compound **6** and Rose Bengal in water and dioxane (50 μM). Light irradiation (520 nm, 0.5 mW, 60 s). Values presented as means±SD (n=3). P values obtained from one-way ANOVA (*** for p<0.001, ns for p>0.05).

First, we measured the singlet oxygen generated by compound **6** upon light irradiation in organic solvents covering a broad range of dielectric constants. As reported by Nagano and colleagues 38, these experiments allow comparison of the behaviors of fluorophores and PS in conditions mimicking different subcellular environments (Figure 3A). Notably, compound **6** generated low levels of singlet oxygen in aqueous media while its production was 5-fold higher in dioxane, a solvent that has a dielectric constant similar to that found in lipophilic cell membranes (Figure 3B) 39, 40. In contrast, the same experiments using the polarity-insensitive Rose Bengal (Figure 3B) showed smaller and non-significant differences (i.e., ~1.5-fold) in singlet oxygen generation between dioxane and water (Figure 3B and Figure S9). Although the exact mechanisms behind these differences in the production of singlet oxygen need further investigations, similar NBD structures have been reported to show reduced fluorescence due to the formation of hydrogen bonding interactions in aqueous media 41, which might quench the excited state and prevent transition to a triplet excited state in the case of benzoselenadiazoles. The accumulation of PS in non-polar regions of the cell (e.g., membranes or envelopes) can favor lipid peroxidation, which ultimately damages the integrity of the cell wall 42. Therefore, these results are in agreement with our observation that the vancomycin-directed localization of nitrobenzoselenadiazoles in the cell envelope of Gram-positive bacteria (Figure 2A) produces enough singlet oxygen to cause cell death, whereas the same PS lacking the envelope-targeting unit (compound **4**) fail to behave as a phototoxic agent. Altogether, nitrobenzoselenadiazole-based PS display strong environmental sensitivity and enhanced production of singlet oxygen in hydrophobic environments, making them valuable scaffolds for the design of PDT agents that target non-polar regions of the cell.

### Nitrobenzoselenadiazole antimicrobial agents can ablate drug-resistant pathogens

In view of the ability of compound **6** to generate singlet oxygen in bacterial cell walls and ablate Gram-positive *S. aureus*, we further examined its capacity to act as a PDT agent for other drug-resistant pathogens, including clinical isolates. We envisaged that the vancomycin moiety of compound **6** would be an effective vector to deliver the PS to the peptidoglycan chains of different Gram-positive bacteria independently of their resistance pattern. This is because known resistance mechanisms prevent vancomycin activity but not localization to the cell envelope. One important advantage of this approach over conventional antibiotic treatments is that a single PDT agent may be able to kill non-resistant and resistant Gram-positive bacteria, being applicable to multiple hospital-acquired infections and reducing antibiotic usage.

First, we examined the targeting capabilities (by fluorescence microscopy) and PDT activity (by CFU counting following aPDT) of compound **6** in a panel of bacterial pathogens commonly found in nosocomial infections 43. These included multi-drug resistant *Staphylococci* (e.g., MRSA), and the vancomycin-resistant* E. faecalis* and *E. faecium,* which exhibit high resistance around 50-100 µg mL^-1^ (34-68 µM), and >100 µg mL^-1^ (>68 µM), respectively (Figure S10). Furthermore, the *E. faecium* strain showed high resistance to teicoplanin (>100 µg mL^-1^ or >58 µM, Figure S12) indicating VanA mediated resistance, whereas *E. faecalis* was teicoplanin sensitive, and thus exhibited VanB mediated resistance. Of note, VanA and VanB are the dominant forms of clinically isolated VRE.

**Figure 4:**
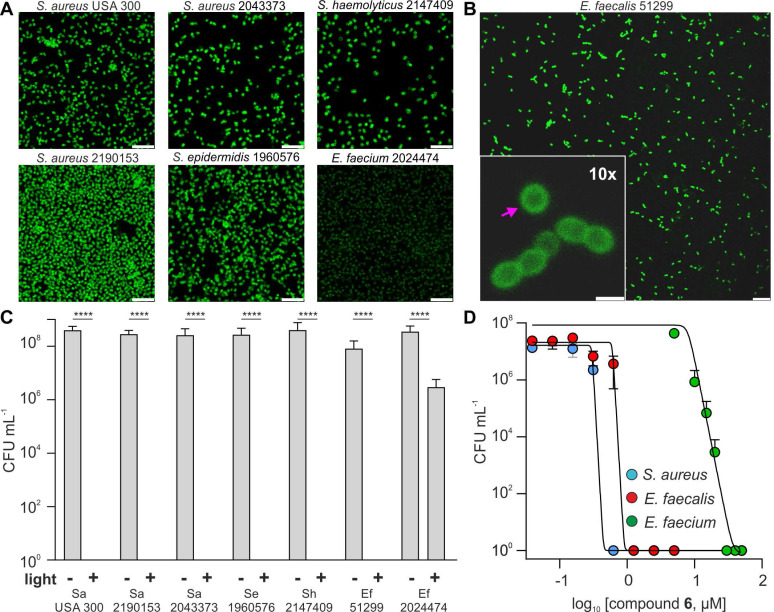
** Compound 6 ablates a wide range of drug-resistant Gram-positive bacteria.** A) Representative fluorescence confocal microscopy images of multiple drug-resistant Gram-positive bacteria after incubation with compound **6** (100 μM). Excitation wavelength: 488 nm. Scale bars: 10 μm. B) Representative fluorescence confocal microscopy images of *E. faecalis* after incubation with compound **6** (100 μM). Excitation wavelength: 488 nm. Emission: 520-650 nm. The pink arrow indicates the preferential localization of compound **6** in the cell envelope. Inset scale bar: 10 μm, right scale bar: 1 μm. C) Cytotoxicity analysis of compound **6** (5 μM) with and without light irradiation (470 nm, 44 mW cm^-2^, 20 min) of drug-resistant bacteria panel (Figure S8 further strain details). Data presented as means±SD (n=3). D) Dose-dependent phototoxicity of compound **6** in *S. aureus*, *E. faecalis* and* E. faecium.* Light irradiation (470 nm, 44 mW cm^-2^, 20 min). Values presented as means±SD (n=3). P values obtained from one-way ANOVA (**** for p<0.0001).

Fluorescence microscopy confirmed that compound **6** bound to the cell envelope of all Gram-positive bacteria strains (Figures 4A and 4B). VRE can replace D-ala for D-lactate in their peptidoglycan chains 36; however, this modification did not hinder the accumulation of compound **6** within the bacterial cell envelope. Next, we evaluated the phototoxicity of compound **6** in all bacterial strains under the same experimental conditions at a working concentration that had proven efficacious in non-resistant *S. aureus* (i.e., 5 μM). We observed that compound **6** was able to kill all strains, with the exception of *E. faecium* (Figure 4B). Further investigation showed that *E. faecium* could be fully eradicated within 20 min after incubation with higher concentrations of compound **6** (i.e., 20-30 µM) and illumination (Figure 4C). Notably, the PDT-MIC of compound **6** for the *E. faecalis* was ~1 µM, which correlates with the higher vancomycin resistance found in *E. faecium* over *E. faecalis* (Figure 4D and Figure S11)*.* Furthermore, we found that the tested strain of *E. faecium* also exhibited high levels of resistance to different antibiotics (e.g., azithromycin, clindamycin, levofloxacin, methicillin, penicillin and teicoplanin) suggesting that this VRE strain has additional modifications on the cell envelope that may reduce the binding ability of compound **6** (Figure S10, S12) 44. Although this panel is not fully exhaustive, these results demonstrate the widespread applicability of compound **6** to eradicate some of the most intractable multi-drug resistant Gram-positive infections, including those which are categorized as the “highest priority” for the development of alternative treatments, such as MRSA and VRE 45.

Next, we examined the potential off-target phototoxicity of compound **6** in human cells, namely red blood cells (RBCs) and keratinocytes as two examples of off-target cells that could be affected in aPDT treatments. First, we performed haemolysis assays in human RBCs from healthy donors and observed that compound **6** did not cause significant haemolysis before or after illumination (Figure S13). Moreover, we ran cell viability assays in the human keratinocyte cell line HaCaT as a relevant model to test the safety of PDT agents 46. As with RBCs, compound **6** showed no significant toxicity under similar conditions to those inducing photoablation of Gram-positive bacteria (Figure S14).

These results indicate that compound **6** can image and kill multiple strains of Gram-positive bacteria, including multi-drug resistant pathogens. Notably, compound **6** showed PDT-MIC values in the high nanomolar range for vancomycin-sensitive bacteria and low micromolar range for VRE strains, and displayed a good safety profile in human cells, being more effective than many conventional antibiotics against resistant infections and a promising translational PDT agent for the treatment of Gram-positive bacterial infections.

### Compound 6 shows antimicrobial efficacy against *S. aureus* biofilms

Biofilms are complex three-dimensional communities of bacteria embedded within an extracellular matrix 47. They are a major cause of persistent infections, particularly associated with indwelling medical devices and chronic wounds. Biofilms allow bacteria to evade phagocytosis by immune cells and can act as a reservoir for antibiotic resistance gene transfer 48. The composition of biofilms leads to high levels of antibiotic, often exhibiting a 10-1,000 fold increase in MIC values due to poor drug penetration and lower metabolic activity 49, 50, thus being refractory to conventional clinical therapies.

PDT has been used successfully to help disturb biofilm structures. For instance, Akhtar and Khan demonstrated the efficacy of aPDT with a sublethal dose (i.e., 2.5 mg mL^-1^) of curcumin to disrupt *S. aureus* biofilms, which were subsequently amenable to complete eradication by leukocytes 51. More recently, the group of Nonell demonstrated that methylene blue-based PDT could kill different resistant strains of *E. coli* in both planktonic and biofilm states; however, they also observed that the phototoxic working concentrations were significantly higher when pathogens were found in biofilms compared to planktonic bacteria 52.

**Figure 5:**
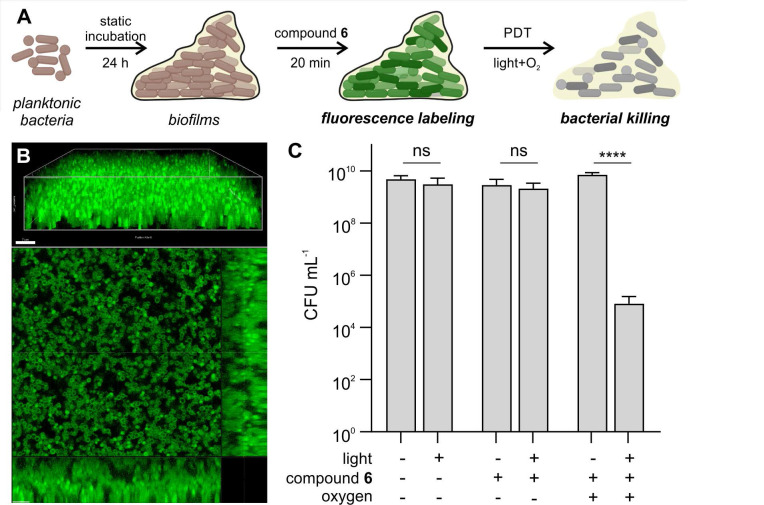
** Compound 6 penetrates biofilms of Gram-positive *S. aureus* and reduces viable bacterial counts following illumination.** A) Schematic illustration of biofilm PDT assays. Planktonic bacteria were incubated for 24 h at 37 °C to form biofilms, which were subsequently incubated with compound **6** and illuminated prior to the assessment of cell viability. B) Representative 3D-rendered confocal scanning laser microscopy image of *S. aureus* biofilms labeled with compound **6** (green, 250 μM) and single slice with orthogonal view. Excitation wavelength: 488 nm. Emission: 495-560 nm. Scale bars: 7 µm. C) Cytotoxicity analysis of biofilms treated with compound **6** (250 μM) before and after light irradiation (470 nm, 44 mW cm^-2^) with and without supplemental oxygen. Data presented as means±SD (n=3). P values obtained from one-way ANOVA (ns for p>0.05, **** for p<0.0001).

For this purpose, we set up a *S. aureus* biofilm model, where bacteria were under static incubation for 24 h to form tightly packed biofilms with a depth over 15 µm (Figure 5A). Notably, compound **6** was able to penetrate throughout the entire biofilm and fluorescently label individual bacterial cells (Figure 5B). Higher concentrations of compound **6** were necessary for labeling biofilms when compared to the planktonic bacteria; yet this was not unexpected because biofilms display 100-fold higher bacterial CFU counts than planktonic experiments. Despite the fluorescent labeling of compound **6** throughout the biofilms, initial attempts in carrying out PDT did not render significant decreases in bacterial viability, likely due to the hypoxic environment found in biofilms. Therefore, we decided to supplement the biofilms with oxygen bubbled into the media 53 ,54, which restored the antimicrobial efficacy of compound **6** and caused over 99% reduction in viable CFU after illumination (Figure 5C). These results indicate that compound **6** can penetrate biofilms to both label and ablate bacterial cells, holding promise as a new theranostic tool to detect and eradicate bacterial infections.

## Conclusions

In this work, we present nitrobenzoselenadiazoles as environmentally sensitive PS for the straightforward derivatization of bacterial-targeting units and generation of efficient theranostic agents for aPDT. We synthesized and characterized different nitrobenzoselenadiazole conjugates and identified compound **6** as a novel phototheranostic for selective fluorescence imaging and light-controlled killing of Gram-positive bacteria. Compound **6** contained a vancomycin moiety that localized the nitrobenzoselenadiazole PS in close proximity to the bacterial peptidoglycan chains, where it exerted the strongest photodynamic activity. Direct comparative analysis of the singlet oxygen generation between nitrobenzoselenadiazole PS and other commercial PS (e.g., Rose Bengal) proved the environmental sensitivity of the former, and its suitability for the preparation of aPDT agents accumulating in bacterial cell envelopes. In addition to selectivity over Gram-negative bacteria, compound **6** exhibited rapid phototoxicity against Gram-positive pathogens (e.g., complete eradication in less than 30 min) and remarkably low PDT MIC values (i.e., ~300 nM for *S. aureus*). Furthermore, we demonstrated the general applicability of compound **6** as a theranostic agent for Gram-positive antibiotic-resistant bacteria, including a broad panel of multi-drug resistant *Staphylococci* and vancomycin-resistant *Enterococci* pathogens commonly found in nosocomial infections. Finally, we assessed the potential of compound **6** for translational PDT studies. Compound **6** showed good safety profiles in human cells (e.g., red blood cells and keratinocytes) and was able to penetrate *S. aureus* biofilms to fluorescently label bacterial cells and cause 99% reduction in viable CFU after illumination. The versatility and modularity of this chemical platform will speed up the design and synthesis of new phototheranostic agents for refractory bacterial infections.

## Supplementary Material

Supplementary figures, information.

## Abbreviations

AMR: Antimicrobial resistance
aPDT: Antimicrobial photodynamic therapy
CFU: Colony forming units
LB: Luria-Bertani Broth
MIC: Minimum inhibitory concentration
MRSA: Methicillin-resistant *S. aureus*
MSSA: Methicillin-sensitive *S. aureus*
PDT: Photodynamic therapy
PS: Photosensitizer
ROS: Reactive Oxygen Species
UHPT: Universal hexose phosphate transporter
VR: Vancomycin resistance
VRE: Vancomycin-resistant *Enterococci*
ε: Extinction coefficient
λ: Wavelength
φ_Δ_: Singlet oxygen quantum yield
