# Supplementary material for: Environmentally sensitive photosensitizers enable targeted photodynamic ablation of Gram-positive antibiotic resistant bacteria
Source: Theranostics. 2023 Jun 26;13(11):3814–25. doi: 10.7150/thno.84187 (PMC10334829; doi:10.7150/thno.84187)
Supplement: Supplementary file 1 — Supplementary figures, information. [file thnov13p3814s1.pdf]

Electronic Supporting Information

Environmentally sensitive photosensitizers enable targeted photodynamic ablation of Gram-positive resistant bacteria

Table of contents

1. Supplementary figures
2. Chemical synthesis and characterization
3. NMR spectra
4. Supplementary references

Supplementary figures

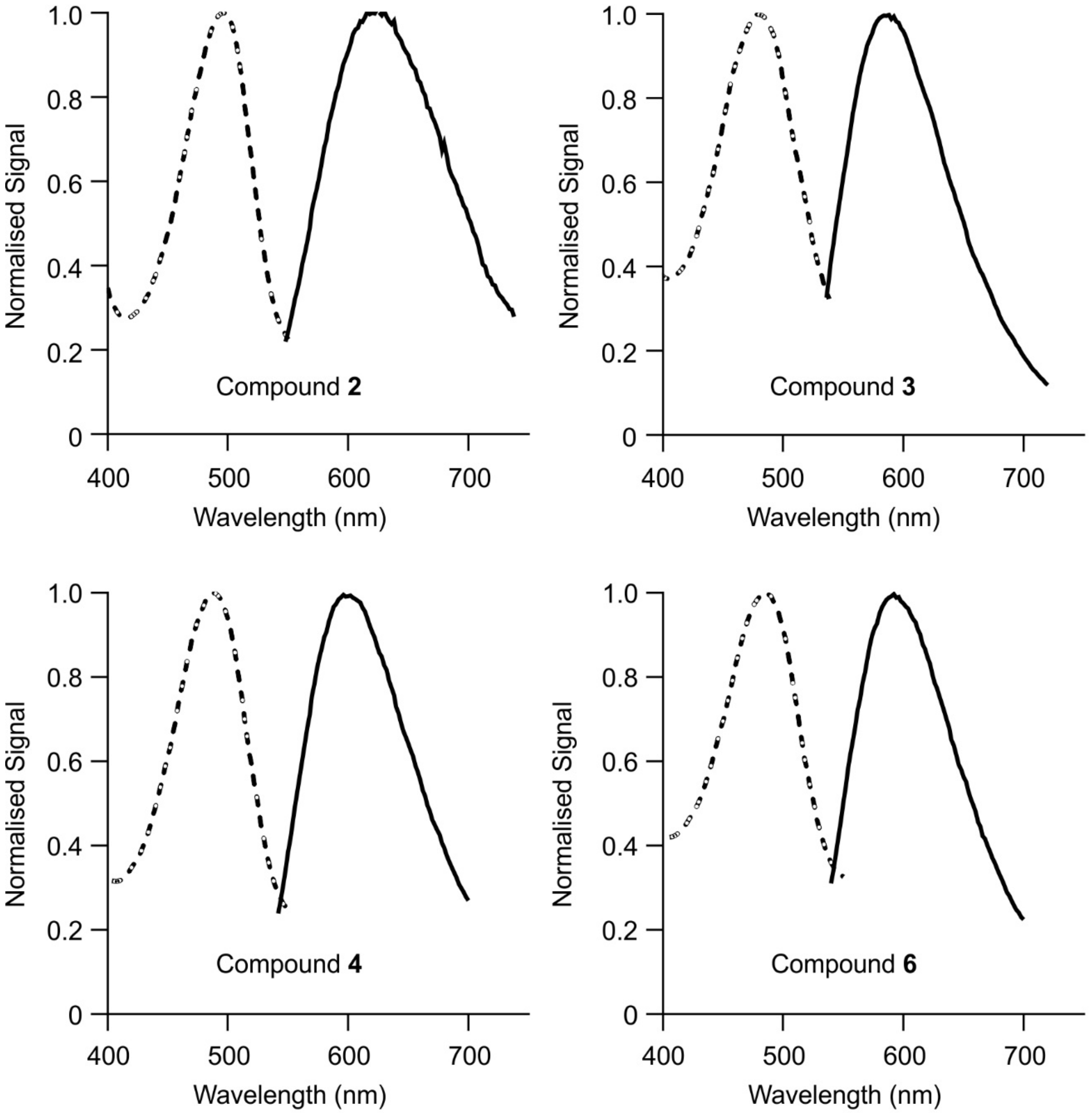

Figure S1. Absorbance and emission spectra of compounds 2, 3, 4 and 6. Compounds were dissolved in EtOH (200 µM) and absorbance spectra (dashed lines) and emission spectra (solid lines) were recorded with excitation wavelength as 480 nm. Data presented as means from 3 independent experiments.

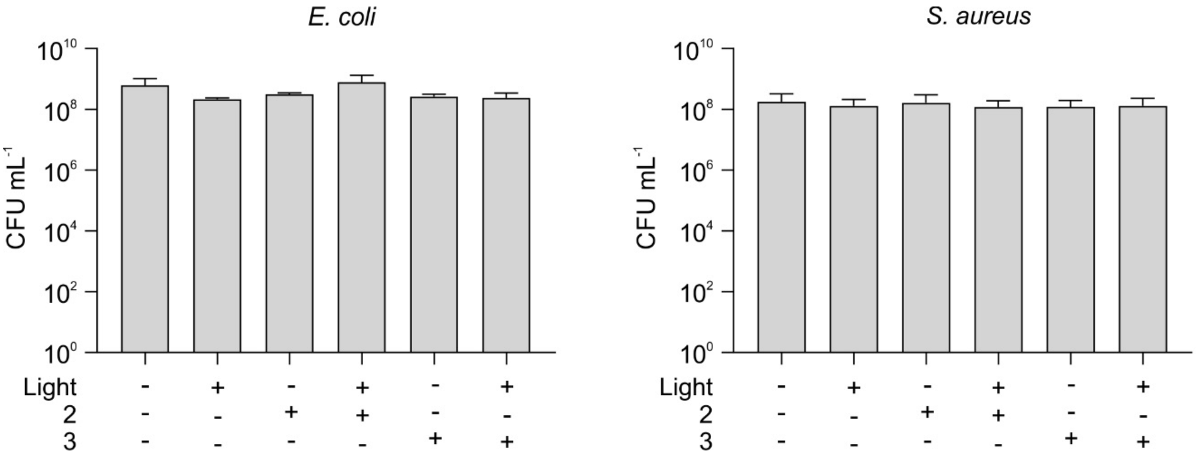

Figure S2. Phototoxicity assays of compounds 2 and 3 in bacterial cells. Phototoxicity of compounds **2** and **3** (5 μM) in *S. aureus* and *E. coli* with and without light irradiation (470 nm, 44 mW cm^{-2}, 20 min). Data presented as means±SD (n=3).

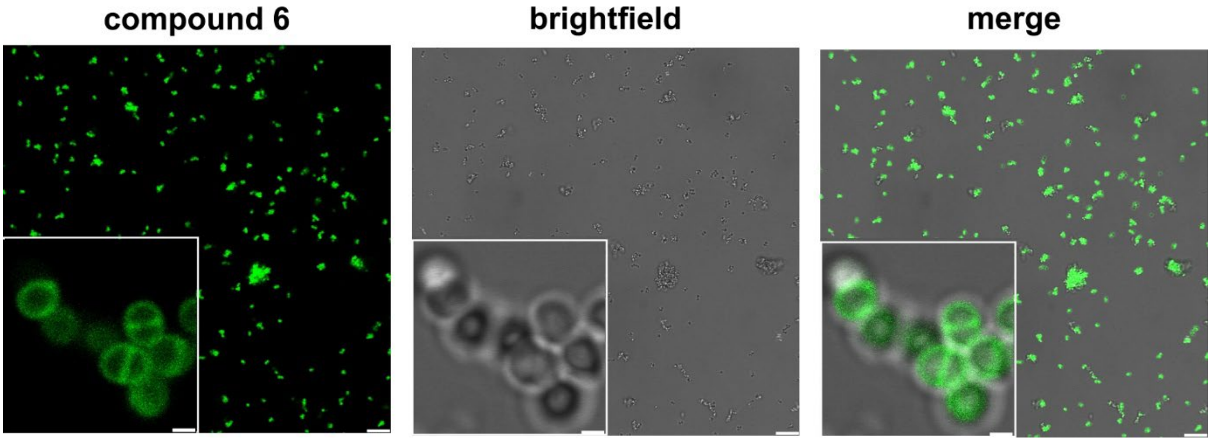

Figure S3. **Confocal imaging of *S. aureus* after staining with compound 6**. Representative brightfield and fluorescence confocal microscopy images of *S. aureus* cells after incubation with targeted PS (compound **6**, 100 μM, green). Excitation wavelength: 488 nm. Emission: 510-650 nm. Scale bar: 10 μm. High-magnification images of *S. aureus* incubated with compound **6**. Scale bar: 1 μm.

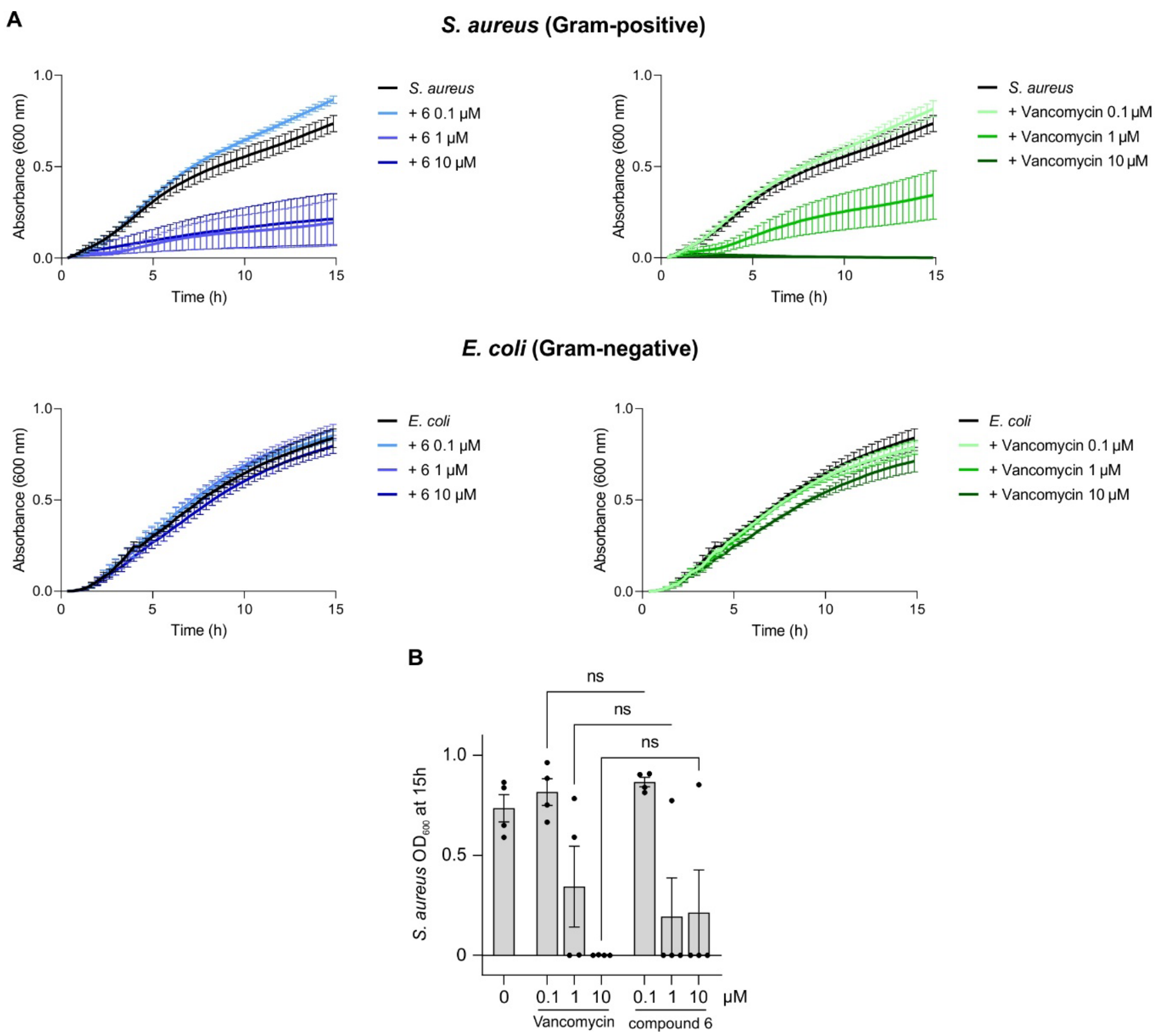

Figure S4. Compound 6 shows similar dark toxicity in *S. aureus* to unlabeled vancomycin. A) Growth kinetics of *S. aureus* and *E. coli* in the dark and in the presence of increasing concentrations of vancomycin (green lines) and compound **6** (blue lines). Growth rates were determined by measuring absorbance at 600 nm. Data presented as means±SEM (n=4). B) Growth values of *S. aureus* after 15 h in the presence of increasing concentrations of vancomycin and compound **6**. P values obtained from one-way ANOVA (ns for p>0.05).

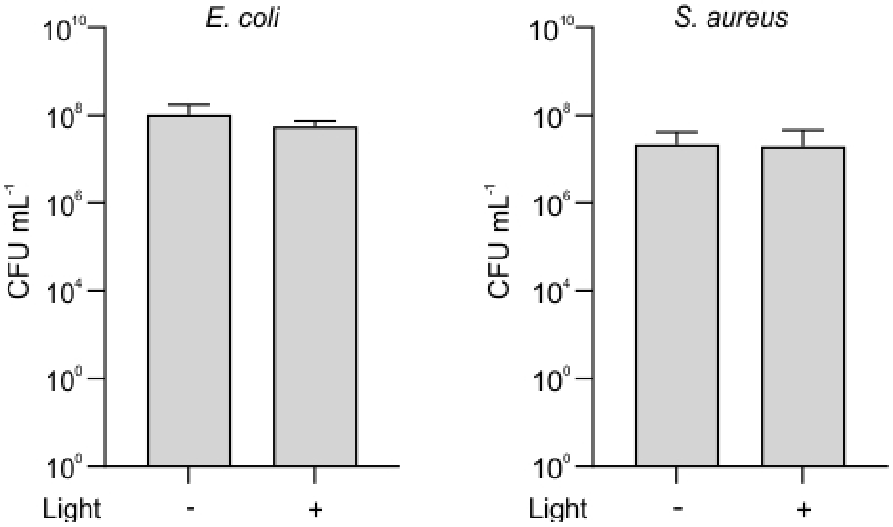

Figure S5. **Standard illumination dosages do not kill *E.coli* or *S. aureus* cells**. Phototoxicity assays of light alone in S. aureus and E. coli (470 nm, 44 mW cm^{-2}, 20 min). Data presented as means±SD (n=3).

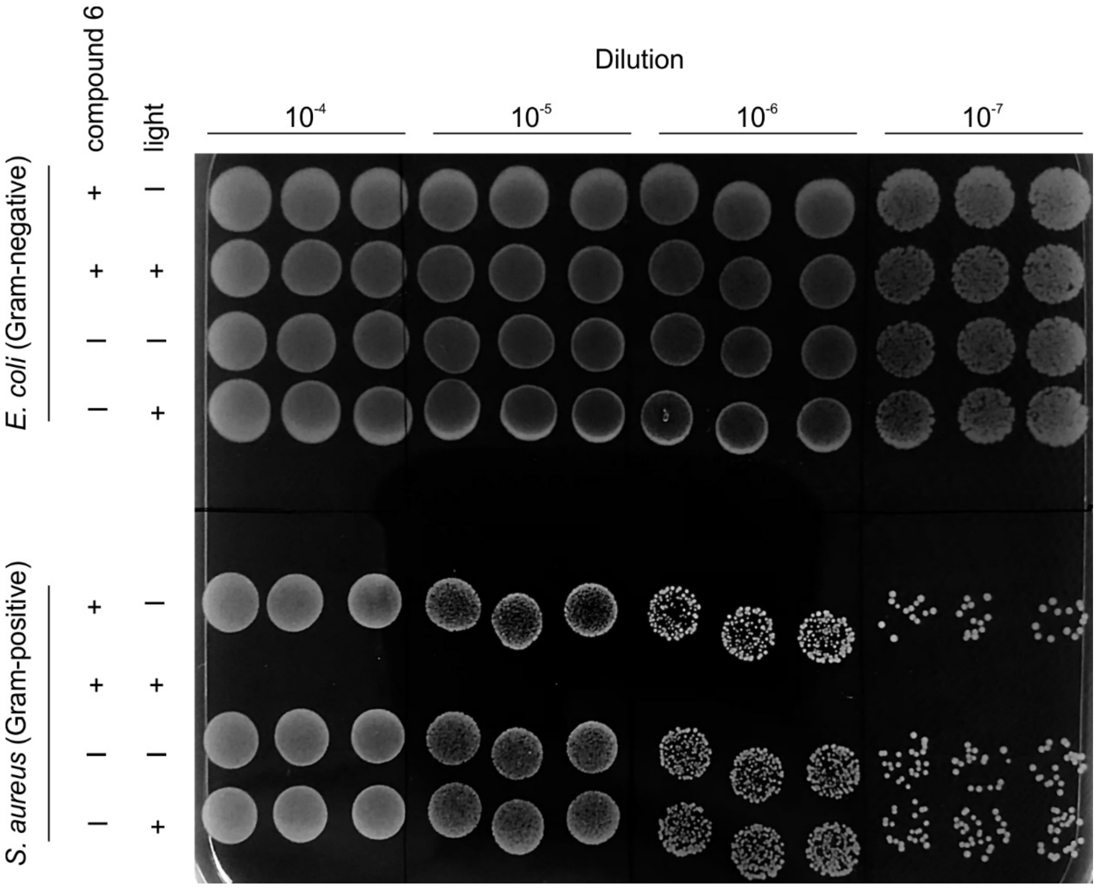

Figure S6. Phototoxicity analysis of compound 6 against of *E. coli* and *S. aureus*. Representative images of plates including *E. coli* and *S. aureus* after incubation with and without compound **6** (5 μM) and with and without illumination (470 nm, 44 mW cm^{-2}, 20 min).

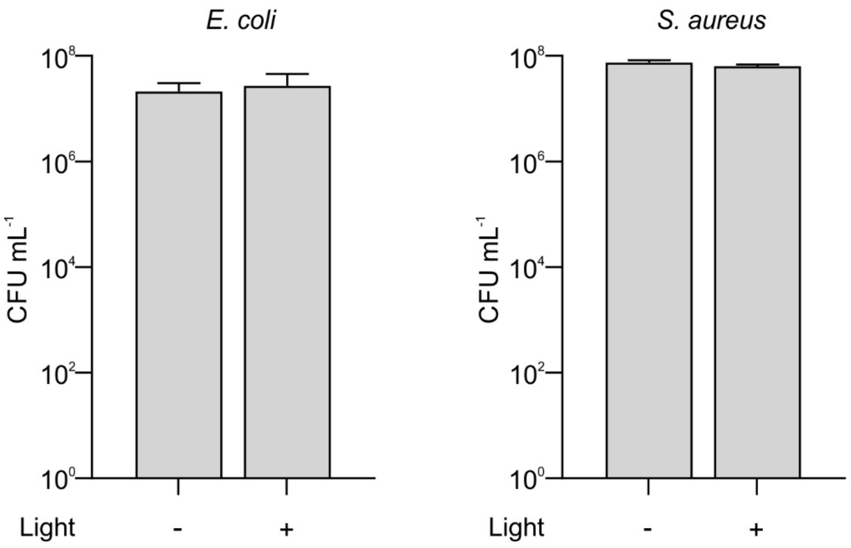

Figure S7. Phototoxicity analysis of unlabeled vancomycin in *E. coli* and *S. aureus*. Phototoxicity assays of vancomycin (5 μM) in *E. coli* and *S. aureus* bacteria with and without light irradiation (470 nm, 44 mW cm^{-2}, 20 min). Data presented as means±SD (n=3).

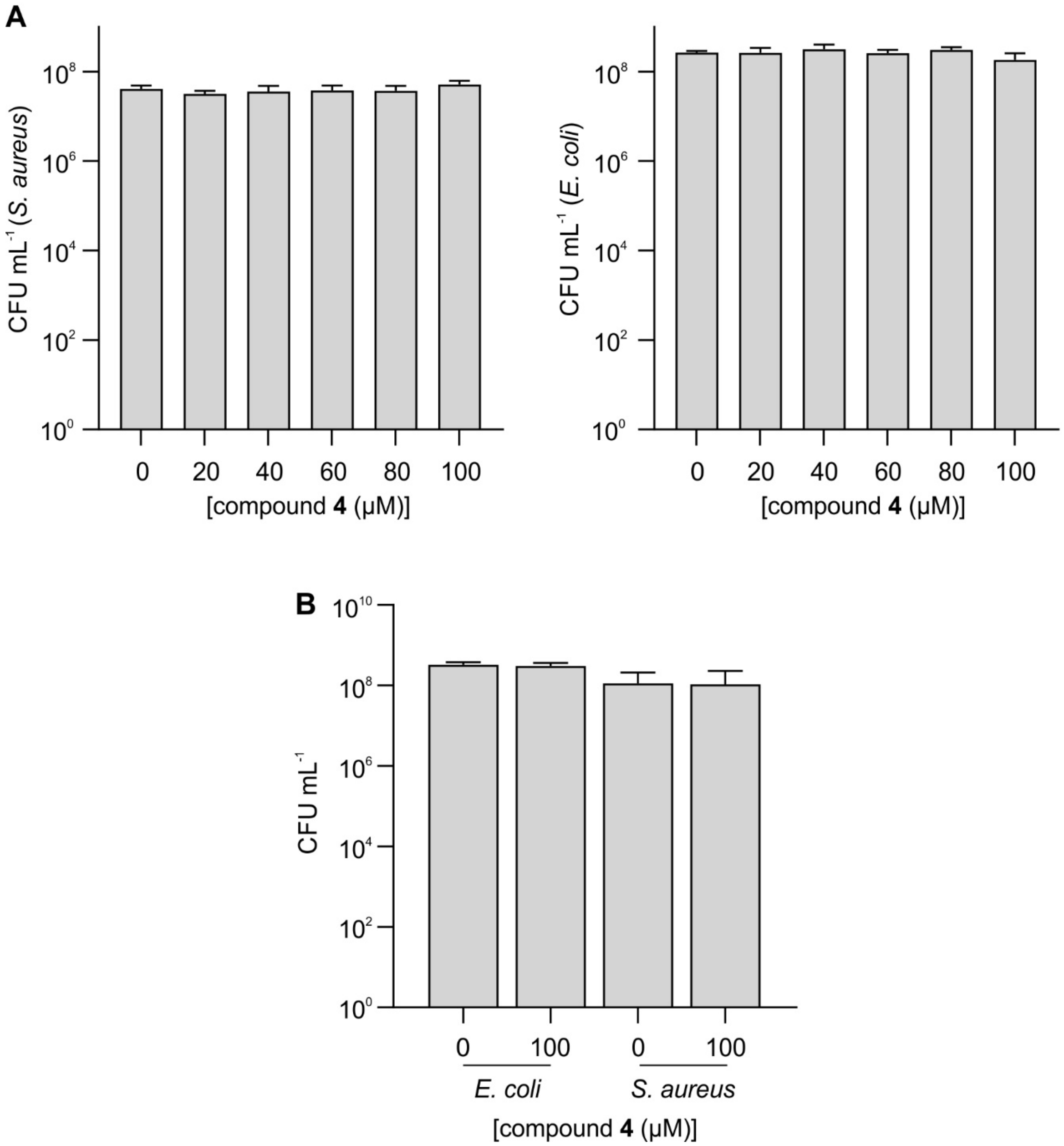

Figure S8. **Phototoxicity analysis of compound 4.** A) Phototoxicity of compound **4** (0-100 μM) in *S. aureus* and *E. coli* bacteria with light irradiation (470 nm, 44 mW cm^{-2}, 20 min). B) Phototoxicity of compound **4** (100 μM) in *S. aureus* and *E. coli* bacteria with light irradiation (470 nm, 44 mW cm^{-2}, 20 min). Data presented as means±SD (n=3).

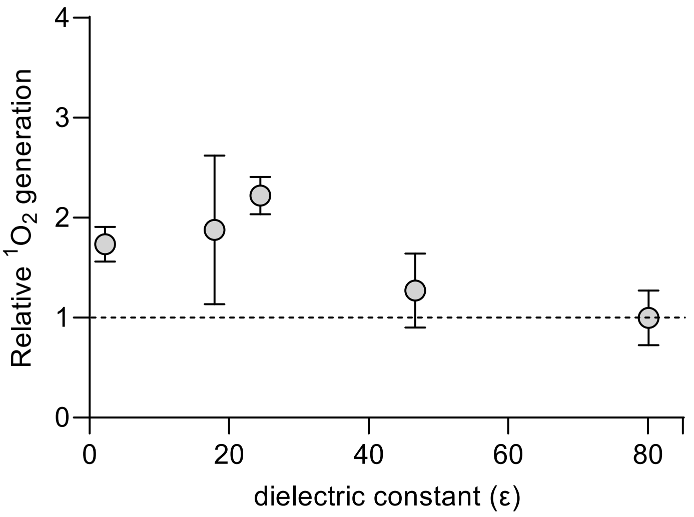

Figure S9. Singlet oxygen generation by Rose Bengal in different organic solvents. Singlet oxygen generation (as measured by changes in absorbance of 1,3-diphenylisobenzofuran) by the photosensitizer Rose Bengal after light irradiation (520 nm, 0.5 mW, 60 s) in solvents of varying dielectric constants. Values normalized to those obtained in H_2O and presented as means±SEM (n=3).

species	strain	antibiotic					
		A	C	L	M	P	V
S. aureus	USA 300				×		
S. aureus	2190153			×	×	×	
S. aureus	2043373			×	×	×	
S. epidermidis	1960576	×	×			×	
S. haemolyticus	2147409	×				×	
E. faecium	2024474	×	×	×		×	×
E. faecalis	51299						×

Figure S10. Antibiotic resistance in the bacterial strains tested in this study. A: azithromycin, C: clindamycin, L: levofloxacin, M: methicillin, P: penicillin, V: vancomycin. Resistance defined for MIC values over 16 μg mL^{-1} and reported by ATCC and IHMA culture collections.

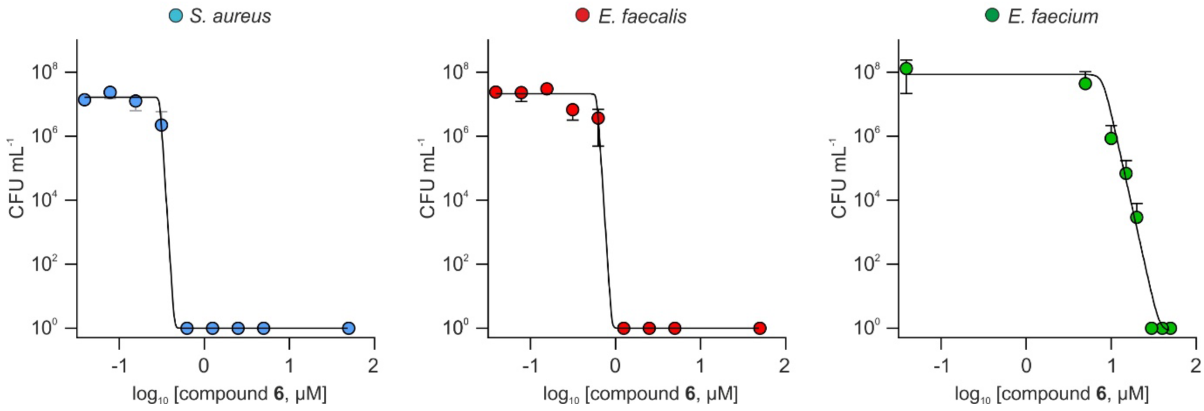

Figure S11. Dose dependent phototoxicity of compound 6 against *S. aureus*, *E. faecalis* and *E. faecium*. Bacterial cells were incubated with increasing concentrations of compound **6** and irradiated with light (470 nm, 44 mW cm^{-2}, 20 min) prior to CFU counting. Data presented as means±SD (n=3).

species	strain	[vancomycin] μg mL^{-1}							
		0	5	10	20	30	40	50	100
S. aureus	USA 300	✓	×	×	×	×	×	×	×
S. aureus	2190153	✓	×	×	×	×	×	×	×
S. aureus	2043373	✓	×	×	×	×	×	×	×
S. epidermidis	1960576	✓	×	×	×	×	×	×	×
S. haemolyticus	2147409	✓	×	×	×	×	×	×	×
E. faecium	2024474	✓	✓	✓	✓	✓	✓	✓	✓
E. faecalis	51299	✓	✓	✓	✓	✓	✓	✓	×

species	strain	[teicoplanin] μg mL^{-1}							
		0	5	10	20	30	40	50	100
S. aureus	USA 300	✓	×	×	×	×	×	×	×
S. aureus	2190153	✓	×	×	×	×	×	×	×
S. aureus	2043373	✓	×	×	×	×	×	×	×
S. epidermidis	1960576	✓	×	×	×	×	×	×	×
S. haemolyticus	2147409	✓	×	×	×	×	×	×	×
E. faecium	2024474	✓	✓	✓	✓	✓	✓	✓	✓
E. faecalis	51299	✓	×	×	×	×	×	×	×

Figure S12. Bacterial viability with increasing concentrations of vancomycin and teicoplanin. Bacterial cells were grown on agar plates containing increasing concentrations of vancomycin and teicoplanin. × = no growth, ✓ =growth

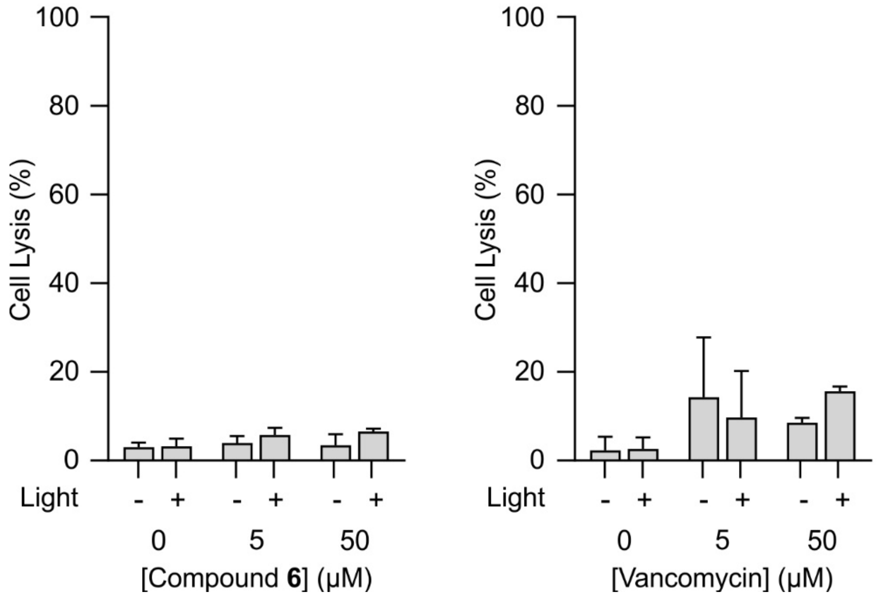

Figure S13. Haemolysis assays in human red blood cells after incubation with compound 6 or unlabeled vancomycin. Compound **6** and vancomycin were added at the indicated concentrations to red blood cells, and haemolysis rates were measured after 1 h with and without light irradiation (470 nm, 44 mW cm^{-2}, 20 min). Data normalised to 100% lysis (by sonication) and 0% lysis (in PBS only), and presented as means±SD (n=3).

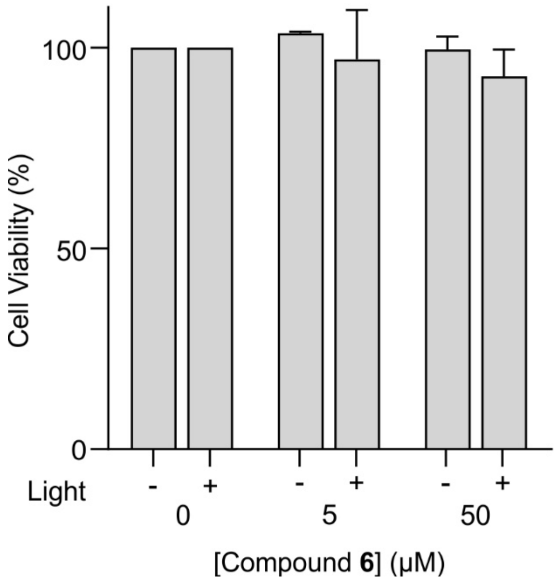

Figure S14. Cytotoxicity of compound 6 in human keratinocytes. Compound **6** was added at the indicated concentrations to HaCaT cells, and cell viability was measured after 24 h with and without light irradiation (470 nm, 10 mW, 1 h). Data normalised to values obtained in 1% DMSO, and presented as means±SD (n=3).

Chemical synthesis and characterization

4-(2-deoxyglucosamine-6-phosphate)-7-nitrobenzo[c][1,2,5]selenadiazole (2)

2-deoxyglucosamine-6-phosphate (42.3 mg, 0.16 mmol) and compound **1**[1] (42.3 mg, 0.17 mmol) were added to ACN (5 mL) at r.t. and $NaHCO_3$ (35.6 mg, 2.44 mmol) in water (5 mL) for 24 h. Volatiles were removed and the aqueous mixture was purified by preparative HPLC (H_2O-ACN with 1% TFA as eluents) to give compound **2** as a red powder (14.6 mg, 18% yield, ~ 1:1 mixture of anomers).

^{1}H NMR (500 MHz, D_2O) δ: 8.68 (m, 2H), 6.63 (d, 9.31 Hz, 1H), 6.58 (d, 9.31 Hz 1H), 5.41 (d, 3.26 Hz, 1H), 4.95 (d, 6.94 Hz, 1H), 4.02 (m, 7H), 3.84 (m, 2H), 3.73 (m, 2H), 3.58 (m, 1H).

^{13}C NMR (75 MHz, DMSO-d6) δ: 152.5, 152.1, 150.3, 135.9, 135.5, 128.8, 127.9, 99.2, 99.1, 95.9, 90.5, 75.5, 75.4, 74.7, 73.1, 71.4, 71.3, 70.8, 70.5, 65.6, 61.9, 58.1.

HRMS (ESI+) m/z calcd. for $C_{12}H_{14}N_4O_{10}PSe$ $[M+H]^+$: 484.9618; found, 484.9606.

4-(amoxicillin)-7-nitrobenzo[c][1,2,5]selenadiazole (3)

Amoxicillin (25 mg, 0.07 mmol) and compound 1 (17 mg, 0.07 mmol) were dissolved in DMSO (2 mL) and stirred at r.t. for 16 h. The solution was diluted in water (28 mL) and lyophilized. The crude mixture was purified by normal-phase flash chromatography (DCM:MeOH 9:1 including 1% formic acid) to give compound **3** as a red powder (23.0 mg, 57% yield).

^{1}H NMR (500 MHz, DMSO-d6) *δ*: 13.21 (s, 1H), 9.52 (s, 1H), 9.19 (d, 7.8 Hz, 1H), 8.55 (d, 8.7 Hz, 1H), 7.76 (d, 7.1 Hz, 1H), 7.35 (d, 8.7 Hz, 2H), 6.73 (d, 8.7 Hz, 2H), 6.13 (d, 8.8 Hz, 1H), 5.62 (dd, 7.8 Hz, 4.0 Hz, 1H), 5.58 (d, 7.0 Hz, 1H), 5.41 (d, 4.0 Hz, 1H), 4.20 (s, 1H), 1.53, (s, 3H), 1.41 (s, 3H).

^{13}C NMR (75 MHz, DMSO-d6) *δ*: 173.4, 170.1, 169.3, 157.9, 152.3, 151.9, 145.7, 134.9, 130.2, 128.6, 127.4, 116.0, 99.0, 71.0, 67.4, 64.2, 58.6, 58.4, 30.7, 27.1.

HRMS (ESI+) m/z calcd. for $C_{22}H_{20}N_6O_7SSe$ $[M+H]^+$: 593.0352; found, 593.0344.

4-((4'-hydroxymethyl)benzylamino)-7-nitrobenzo[c][1,2,5]selenadiazole (4)

Compound **1** (120 mg, 0.5 mmol) was added to a solution of 4-aminomethylbenzyl alcohol (75 mg, 0.55 mmol) in 5 mL EtOH:DCM (4:1). The reaction mixture was stirred at r.t. for 2 h. Volatiles were then removed, and the crude mixture purified by normal-phase flash chromatography (gradient hexane → EtOAc) to give compound **4** as red powder (156 mg, 86% yield).

^{1}H NMR (500 MHz, DMSO-d6) *δ*: 9.00 (t, 6.5 Hz, 1H), 8.51 (d, 9.0 Hz, 1H), 7.35 (d, 8.2 Hz, 2H), 7.28 (d, 8.2 Hz, 2H), 6.28 (d, 9.0 Hz, 1H), 5.12 (t, 5.5 Hz, 1H), 4.68 (d, 6.5 Hz, 2H), 4.46 (d, 5.5 Hz, 2H).

^{13}C NMR (75 MHz, DMSO-d6) *δ*: 157.8, 152.5, 152.4, 142.0, 136.5, 135.4, 129.0, 127.3, 127.2, 63.1.

HRMS (ESI+) m/z calcd. For $C_{14}H_{13}N_4O_3Se$ $[M+H]^+$: 365.0147; found, 365.0138.

4-(benzylamino-4-carbaldehyde)-7-nitrobenzo[c][1,2,5]selenadiazole (5)

Compound **4** (72 mg, 0.2 mmol) was stirred in 10 mL of anhydrous DCM under inert atmosphere. Dess-Martin Periodinane (100 mg, 0.24 mmol) was added at once and the reaction was stirred at r.t. for 4 h. The reaction was then quenched by addition of 3 mL 20% aqueous $Na_2S_2O_3$ and 12 mL saturated aqueous $NaHCO_3$ and stirred 30 min at r.t. The reaction mixture was diluted with 20 mL DCM, and the organic phase was separated. The aqueous phase was extracted with DCM (3 × 50 mL). The organic phases were combined, dried over Na_2SO_4, and concentrated under reduced

pressure. The crude mixture was purified by normal-phase flash chromatography (gradient DCM → DCM:MeOH (95:5)) to isolate compound **5** as red powder (70 mg, 97% yield). Compound **5** was not purified and used for the next step.

4-(vancomycin)-7-nitrobenzo[c][1,2,5]selenadiazole (6)

Compound **5** (36 mg, 0.1 mmol) and Vancomycin HCl (100 mg, 0.07 mmol) were dissolved in 2 mL DMF. Diisopropylethylamine (0.04 mL) was then added, and the reaction was stirred at 55°C for 4 h and allowed to cool down to r.t. Sodium cyanoborohydride (21 mg, 0.34 mmol) in 2 mL MeOH and trifluoroacetic acid (0.07 mL) were added to the reaction mixture, which was stirred for 2 h at r.t. Volatiles were removed and the solid obtained was washed with Et_2O (3 × 15 mL). Finally, the crude mixture was purified by preparative HPLC (H_2O-ACN with 1% TFA as eluents) to give compound **6** as red powder (56 mg, 32% yield).

^{1}H NMR (500 MHz, $CDCl_3$) *δ*: 9.60, 9.46, 9.18, 9.11, 9.04, 8.84, 8.66, 8.55, 8.51, 8.12, 7.87, 7.59, 7.48, 7.46, 7.42, 7.37, 7.33, 7.26, 7.21, 7.17, 7.02, 6.78, 6.73, 6.68, 6.43, 6.25, 5.98, 5.77, 5.62, 5.37, 5.29, 5.20, 5.12, 4.94, 4.72, 4.67, 4.48, 4.44, 4.28, 4.20, 3.95, 3.70, 3.58, 3.54, 3.28, 3.17, 2.64, 2.55, 2.07, 1.86, 1.76, 1.71, 1.62, 1.50, 1.24, 1.12, 0.93, 0.87.

HRMS (ESI+) m/z calcd. for $C_{80}H_{85}Cl_2N_{13}O_{26}Se$ $[M+2H]^{2+}$: 897.2425; found, 897.7229.

NMR spectra

Compound **2**

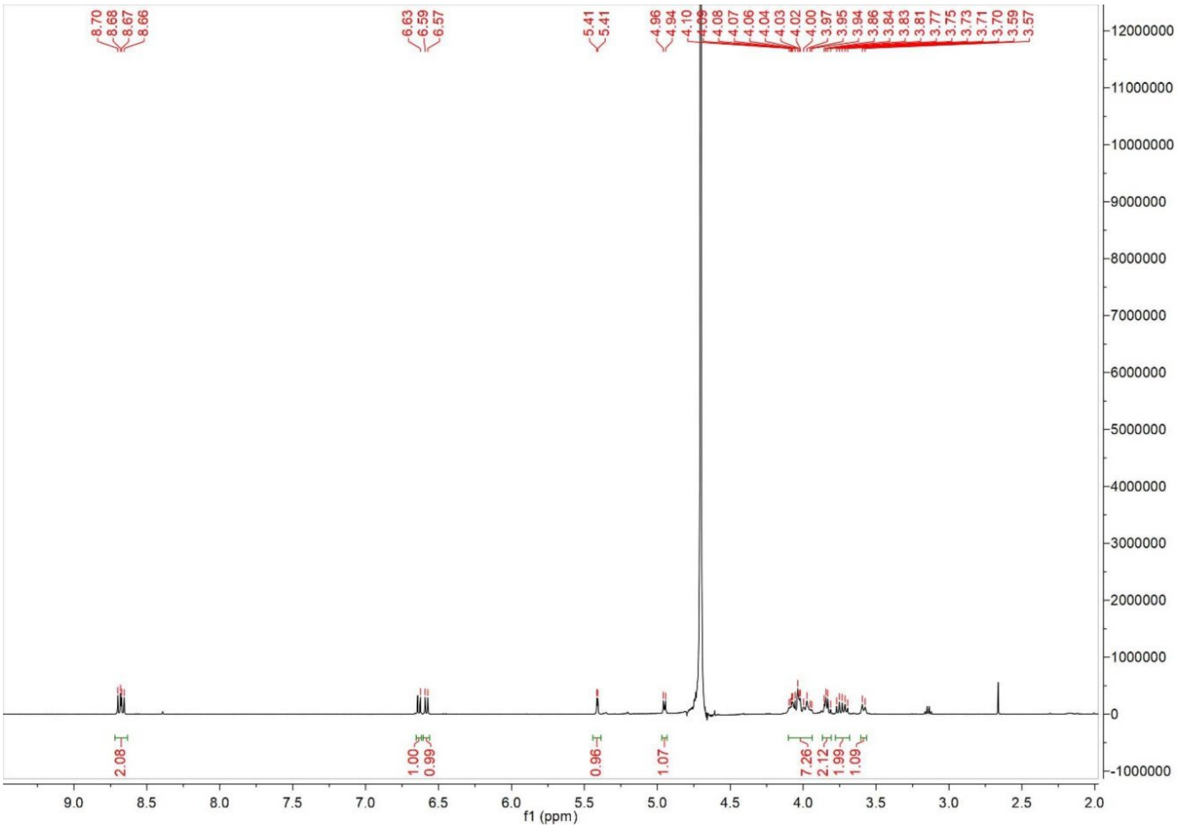

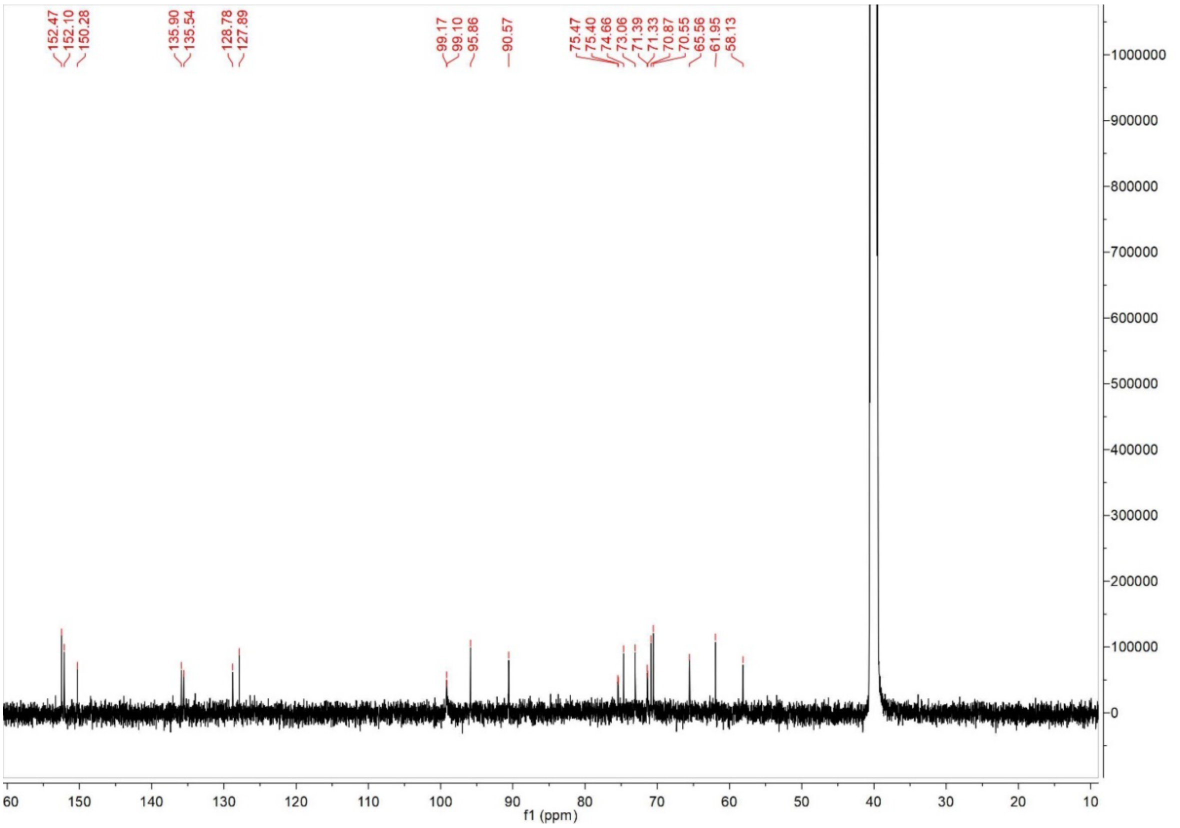

Compound **3**

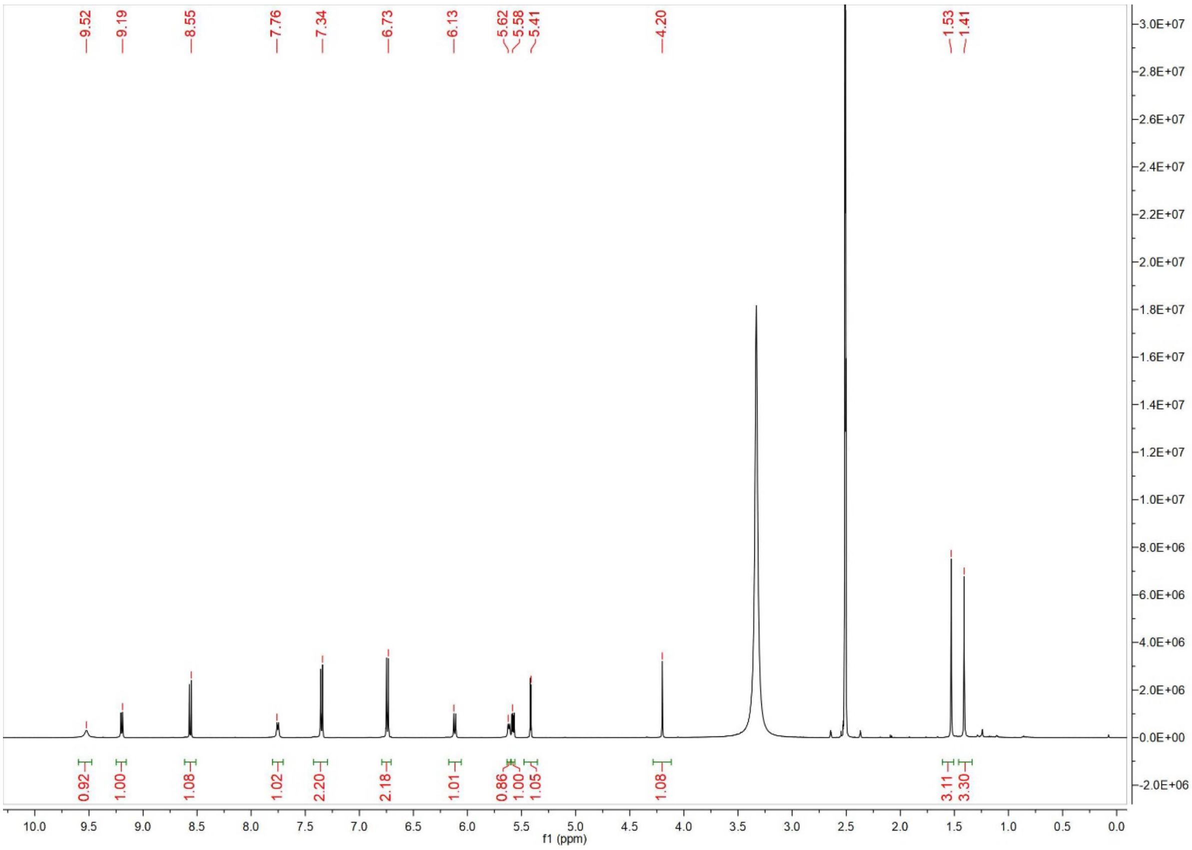
9.52
9.19
8.55
7.76
7.34
6.73
6.13
5.62
5.58
5.41
4.20
1.53
1.41
0.92
1.00
1.08
1.02
2.20
2.18
1.01
0.86
1.00
1.05
1.08
3.11
3.30
f1 (ppm)

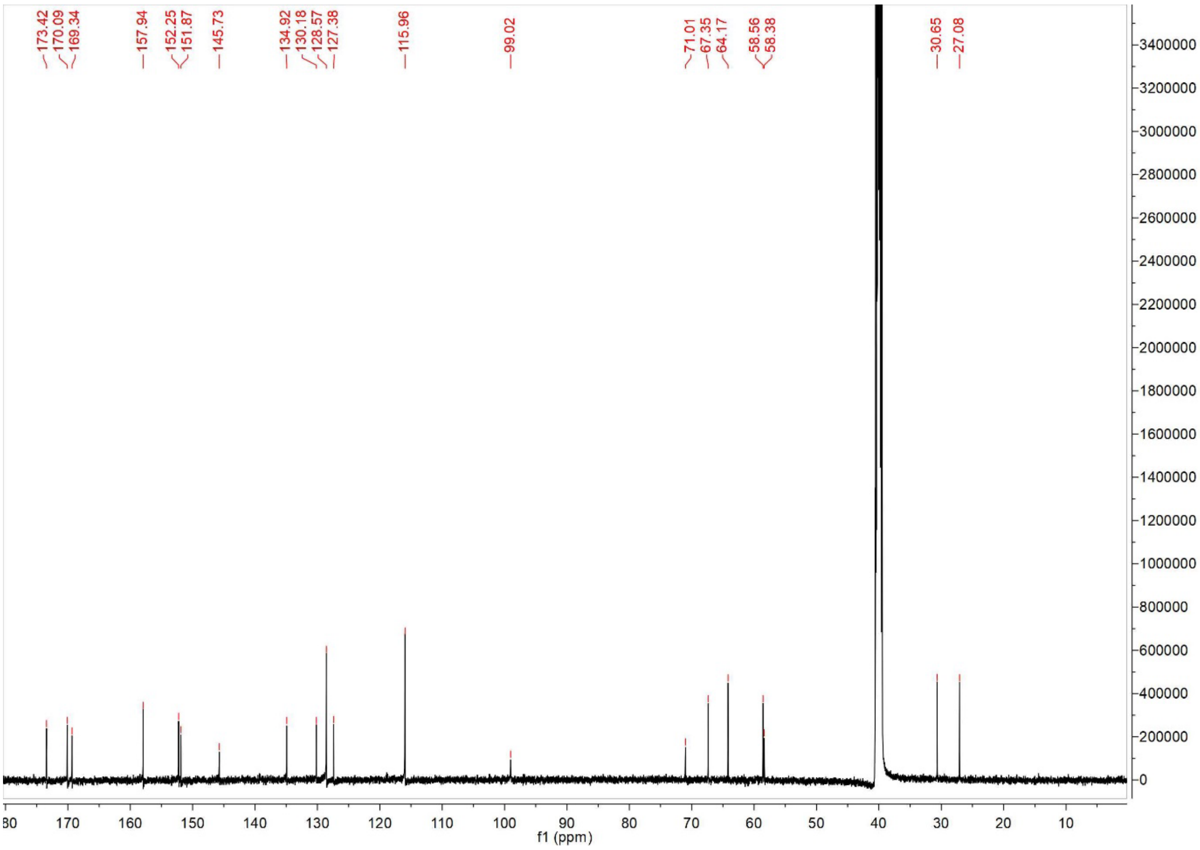
173.42
170.09
169.34
157.94
152.25
151.87
145.73
134.92
130.18
128.57
127.38
115.96
99.02
71.01
67.35
64.17
58.56
58.38
30.65
27.08
f1 (ppm)

Compound **4**

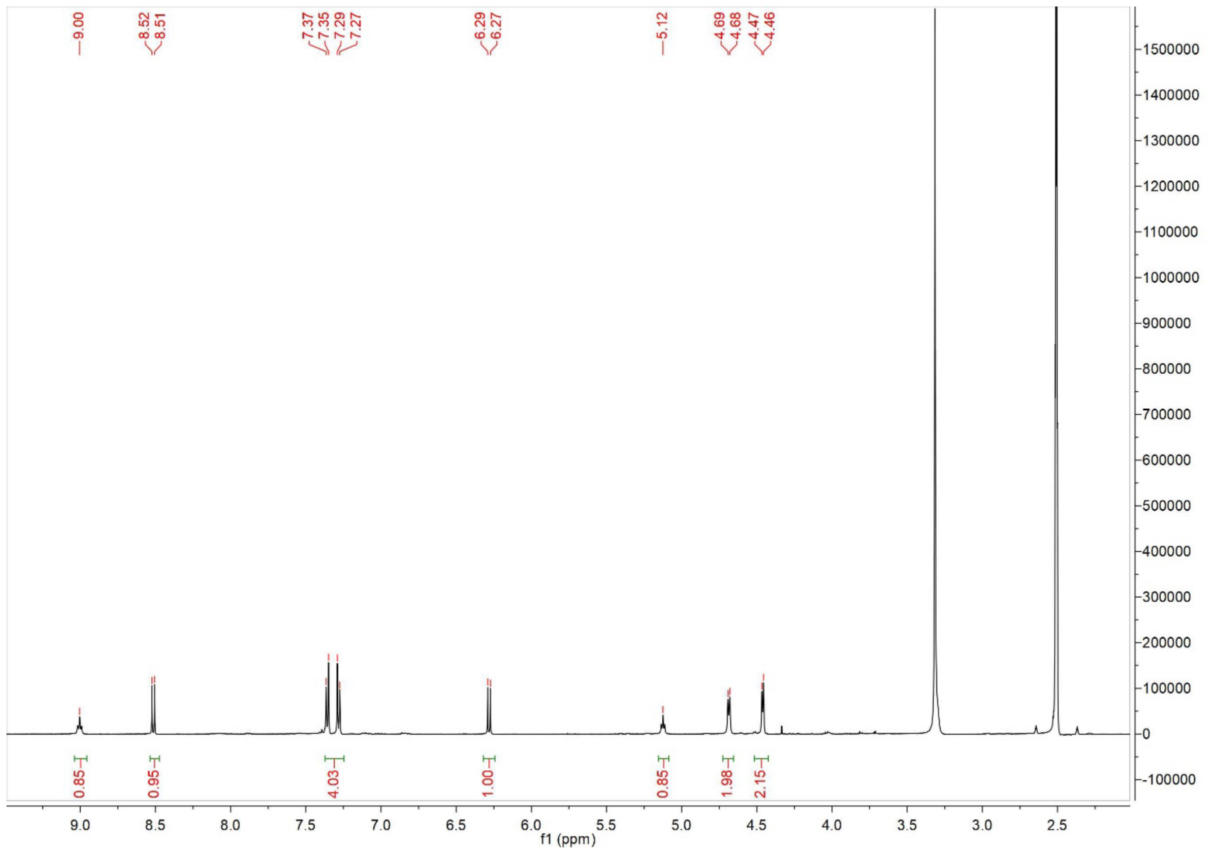
9.00
8.52
8.51
7.37
7.35
7.29
7.27
6.29
6.27
5.12
4.69
4.68
4.47
4.46
0.85
0.95
4.03
1.00
0.85
1.98
2.15
f1 (ppm)

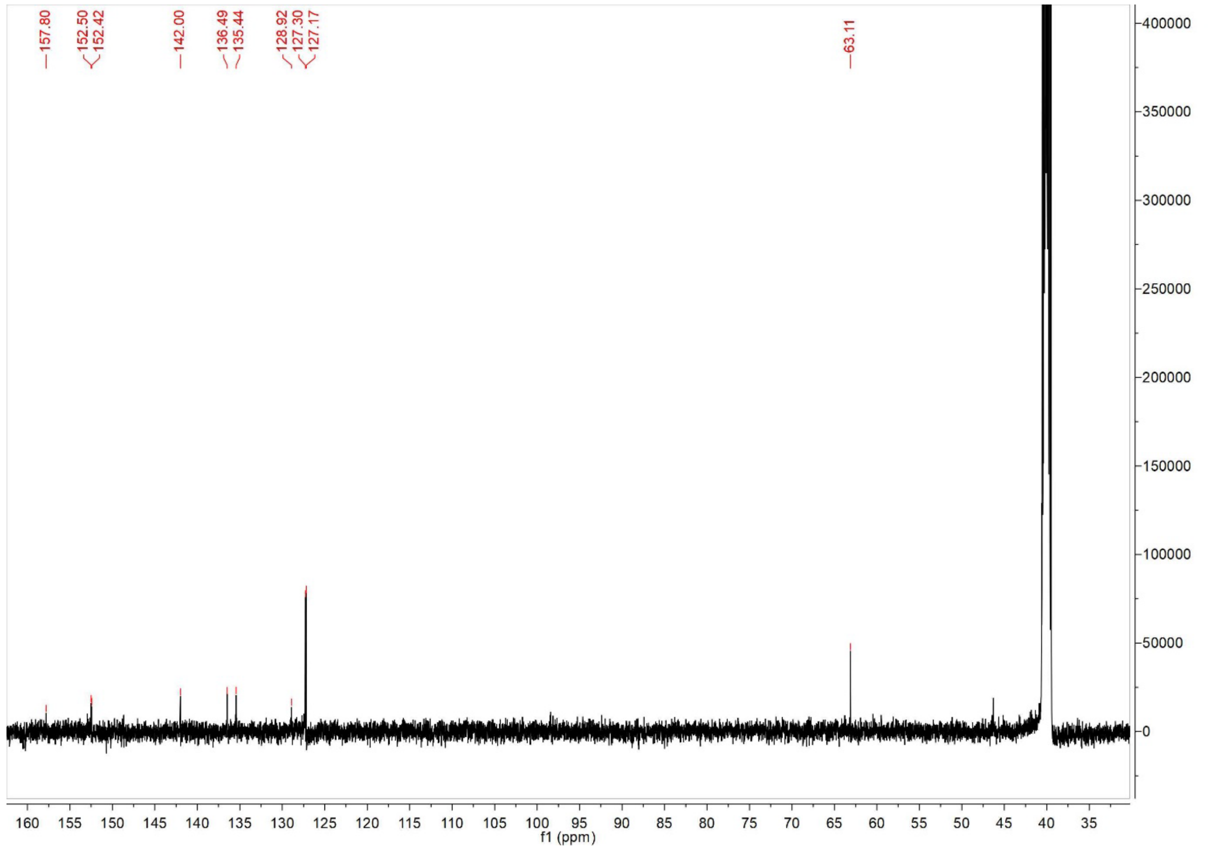
157.80
152.50
152.42
142.00
136.49
135.44
128.92
127.30
127.17
63.11
f1 (ppm)

Compound **6**

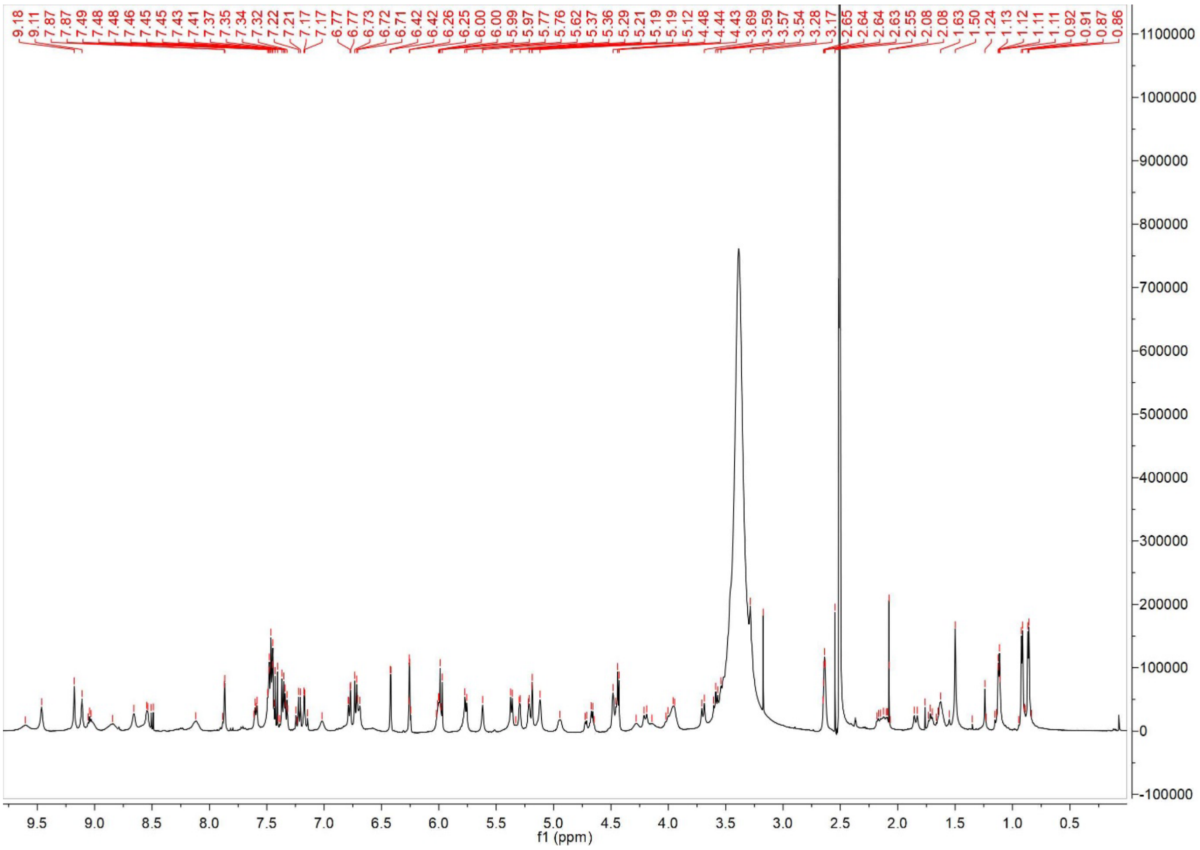

9.18
9.11
7.87
7.49
7.48
7.46
7.45
7.43
7.41
7.37
7.35
7.34
7.32
7.22
7.21
7.17
7.17
6.77
6.77
6.73
6.72
6.71
6.42
6.42
6.26
6.25
6.00
6.00
5.99
5.97
5.77
5.76
5.62
5.37
5.36
5.29
5.21
5.19
5.12
4.48
4.44
4.43
3.69
3.59
3.57
3.54
3.28
3.17
2.65
2.64
2.64
2.63
2.55
2.08
1.63
1.50
1.24
1.13
1.12
1.11
1.11
0.92
0.91
0.87
0.86
1100000
1000000
900000
800000
700000
600000
500000
400000
300000
200000
100000
0
-100000
9.5 9.0 8.5 8.0 7.5 7.0 6.5 6.0 5.5 5.0 4.5 4.0 3.5 3.0 2.5 2.0 1.5 1.0 0.5
f1 (ppm)

Supplementary references

[1] Benson S, Fernandez A, Barth N, de Moliner F, Horrocks M, Herrington CS, et al. Angew Chem Int Ed Engl. 2019; 58: 6911–5.
